# Antiseizure Medications in Development: Novel Mechanisms, Precision Therapy, and the Move Towards Disease Modification

**DOI:** 10.3390/cimb48080830

**Published:** 2026-08-16

**Authors:** William Alves Martins

**Affiliations:** 1 Porto Alegre Epilepsy Surgery Program, Neurology and Neurosurgery Services, Hospital São Lucas, Porto Alegre 90610-000, Brazil; martins.william@pucrs.com; Tel.: +55-51-3320-3218; 2 The Brain Institute, Porto Alegre 90610-000, Brazil; 3 School of Medicine, Universidade Luterana do Brasil (ULBRA), Canoas 92425-900, Brazil; 4 School of Medicine, Pontifícia Universidade Católica do Rio Grande do Sul (PUCRS), Porto Alegre 90160-092, Brazil

**Keywords:** epilepsy, antiseizure medications, sodium channels, persistent current, GABA_A_ receptors, precision medicine, developmental and epileptic encephalopathies, disease modification, antisense oligonucleotides

## Abstract

**Background**: Despite more than 30 licenced antiseizure medications (ASMs), approximately one third of people with epilepsy remain drug-resistant, and developmental and epileptic encephalopathies represent one of the greatest unmet needs in epilepsy therapeutics. The past decade has produced a substantial reorientation of ASM discovery, driven by epilepsy genetics, new disease models, advances in drug screening, and innovative therapeutic modalities. **Objective**: The objective of this study was to review the contemporary clinical-stage pipeline of ASMs with novel or differentiated mechanisms of action, organized by molecular target, while placing recent regulatory successes and instructive failures within the broader transition toward mechanism-based, precision, and potentially disease-modifying therapies. **Findings**: A 2024 pipeline analysis identified more than 200 epilepsy therapies in preclinical or clinical development; at the cutoff of the present literature search (30 June 2026), over 40 compounds had reached phase II or III, with the majority directed at DEEs. Functional-state-selective sodium channel modulation has emerged as a leading conceptual advance supported by converging mechanistic and early clinical evidence, exemplified by relutrigine (PRAX-562), a preferential persistent-current inhibitor for which a regulatory decision is pending in *SCN2A*/*SCN8A*-DEEs, and vormatrigine (PRAX-628), whose large open-label effect was not reproduced in a controlled (blinded) trial. Several of the efficacy figures summarized here derive from congress presentations, interim analyses, or open-label extensions and await full peer-reviewed publication. Parallel advances include the selective Kv7 opener azetukalner; the dual-mechanism benchmark cenobamate; cholesterol-24-hydroxylase inhibition (soticlestat); selective serotonergic agonism (bexicaserin); glutamatergic precision agents (radiprodil); subtype-selective GABA_A_ modulators (darigabat, ganaxolone); and gene-directed therapies (zorevunersen, elsunersen). Pre-symptomatic intervention in tuberous sclerosis complex provides an early, single-trial clinical proof of principle for delaying and reducing the incidence of epilepsy in a genetically defined population; this should not yet be equated with established disease prevention. **Conclusions**: The pipeline reflects an ongoing shift from broad symptomatic agents toward mechanism-led, genotype-matched, and potentially disease-modifying treatments. This shift is tempered by a persistent translational gap between early signals and randomized-trial confirmation, and by the preliminary status of much of the supporting evidence.

## 1. Introduction

Epilepsy affects roughly 50 million people worldwide and remains, for a substantial minority, a refractory and disabling condition. Although the therapeutic armamentarium has more than doubled over the past three decades, the proportion of patients achieving sustained seizure freedom has not improved commensurately: about 30–40% of patients continue to have seizures despite appropriate pharmacotherapy [1]. The probability of attaining control falls sharply with each successive regimen—on the order of 50% with the first ASM, near 12% with the second, and only a few percent thereafter—so that drug resistance, once established, is difficult to overcome with additional broad-spectrum agents [1]. The molecular basis of pharmacoresistance is only partly understood, and most marketed ASMs share a relatively narrow set of mechanisms (sodium-channel blockade, GABAergic enhancement, calcium-channel modulation, and synaptic-vesicle protein binding), limiting the expected benefit of yet another agent acting through familiar pathways [2,3].

Against this backdrop, the past decade has delivered two breakthroughs that meaningfully exceeded prior expectations: cenobamate for common drug-resistant focal epilepsy and fenfluramine for seizures in Dravet syndrome. In terms of seizure freedom and magnitude of seizure reduction, both compare favourably with ASMs introduced in the preceding generation, and both owe part of their success to mechanisms that differ from the standard repertoire; these comparisons are indirect, derived from separate trial programmes with differing populations and comparators rather than from head-to-head studies [4,5]. Their emergence has reframed the field’s ambitions: rather than incremental refinement, developers are pursuing genuinely novel targets, syndrome- and genotype-specific precision treatments, and—most ambitiously—agents that prevent or modify epileptogenesis rather than merely suppressing seizures.

This review surveys the clinical-stage ASM pipeline through the lens of mechanism. After outlining the overall shape of the pipeline, it examines novel agents grouped by molecular target, with particular attention to the most clinically advanced and conceptually distinctive programmes. It then considers gene-directed therapies, the nascent field of antiepileptogenesis, and the recurring translational gap between promising early data and randomized-trial confirmation, closing with a critical appraisal of contemporary development methodology and with implications for clinical practice and future research. Throughout, the emphasis is on how emerging agents may complement, refine, and expand the existing therapeutic armamentarium rather than displace conventional ASMs.

### A Conceptual Framework for the Emerging ASM Pipeline

**Figure 1:**
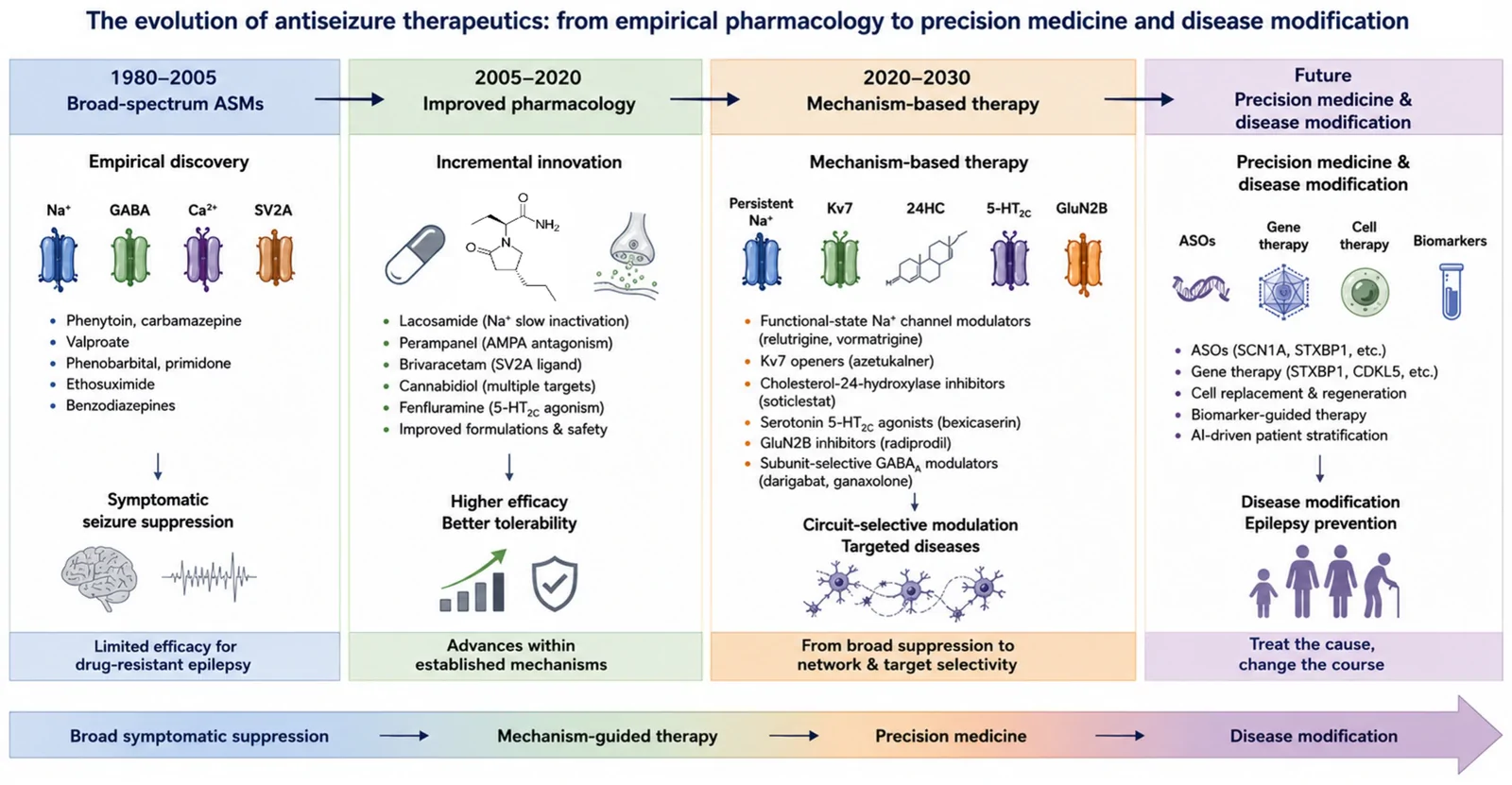
The evolution of antiseizure therapeutics: From empirical pharmacology to precision medicine and disease modification. Conceptual timeline illustrating the evolution of epilepsy therapeutics from empirically developed broad-spectrum antiseizure medications toward mechanism-based pharmacology, precision therapeutics, and disease-modifying strategies. Earlier generations primarily focused on symptomatic seizure suppression through established molecular targets, whereas current and emerging therapies increasingly emphasize circuit-selective modulation, genetically defined epilepsies, biomarker-guided treatment selection, and interventions aimed at altering disease trajectory or preventing epilepsy. Conceptual framework synthesized by the author from the cited literature; the figure contains no original quantitative data, and placement of a therapy within the “disease-modifying” domain denotes the stated objective of the approach rather than demonstrated disease modification. Investigational status shown is current to 30 June 2026. Synthesized from Refs. [2,4,5,6,7,8,9].

Rather than representing a simple expansion in the number of available antiseizure medications, the contemporary pipeline reflects three converging therapeutic paradigms. The first is the development of mechanistically differentiated small molecules that target previously underexploited pathways, such as persistent sodium current inhibition, Kv7 channel activation, and cholesterol-24-hydroxylase inhibition. The second is the emergence of precision therapeutics directed at genetically defined epilepsies, including antisense oligonucleotides, gene therapy, and mutation-specific pharmacology. The third is a shift from symptomatic seizure suppression toward interventions capable of modifying epileptogenesis or altering the natural history of disease. The remainder of this review is organized according to these complementary therapeutic paradigms (Figure 1).

An important caveat applies to the entire framework that follows. The mechanistic categories used in this review reflect the predominant or therapeutically intended actions of each compound rather than absolute molecular exclusivity; several agents have concentration-dependent secondary effects, and their complete pharmacological profiles remain incompletely characterized. Functional selectivity—preferential engagement of a pathological channel state or receptor subtype—is not equivalent to absolute molecular specificity, and selectivity ratios reported in heterologous expression systems need not translate into exclusivity at clinically achievable brain concentrations. Several observations illustrate the point. Lamotrigine, the archetypal sodium-channel blocker, also modulates A-type and other neuronal K^+^ currents and Ca^2+^-sensing cation currents at concentrations above those required for Na_V_ channel inhibition [10]. Cenobamate, whose antiseizure profile is usually described in terms of persistent Na^+^ current inhibition and GABA_A_ modulation, also inhibits several cardiac ion channels, including Ca_V_1.2 and K_V_7.1/minK, in the micromolar range [11]. Conversely, activators of the M-type K^+^ current are not uniformly selective: retigabine, the first-generation K_V_7 opener, potentiates GABA-evoked currents through a direct action on GABA_A_ receptors, with preference for extrasynaptic δ-subunit-containing receptors, and this ancillary mechanism has been proposed to contribute to its clinical spectrum [12,13]. K_V_7 channels are also widely expressed outside the central nervous system, notably in vascular and detrusor smooth muscle, so systemic effects are anticipated for any K_V_7-directed agent [14].

These considerations do not invalidate a mechanism-based organization; on the contrary, they qualify it. Grouping compounds by predominant therapeutic target remains the most informative way to describe a pipeline whose defining feature is target diversification, provided that three points are kept in view. First, ion-channel modulators frequently affect more than one channel family, and receptor-directed compounds may exhibit subtype-dependent, network-level, or off-target effects that are not captured by a single label. Second, pharmacological profiles are provisional and may be revised as additional electrophysiological, receptor-binding, preclinical, and clinical data accumulate; for most investigational agents in this review the published pharmacology comprises a small number of studies, several conducted by the sponsor. Third—and in direct answer to a legitimate question about clinical positioning—agents with incompletely characterized pharmacology are unlikely to displace conventional ASMs in the near term. The more plausible expectation is that they will complement, refine, or expand established therapy: as targeted options in genetically defined syndromes, as mechanistically distinct additions in drug-resistant focal epilepsy, and as tools for testing whether disease modification is achievable at all. Their eventual place in practice will be determined by comparative efficacy, tolerability, durability, accessibility, cost, monitoring burden, drug–drug interactions, and real-world effectiveness rather than by mechanistic novelty alone.

## 2. Methodology and Evidence-Provenance Framework

This is a narrative review conducted with a structured and reproducible search strategy. It is not a systematic review: no protocol was registered, and study selection and data extraction were performed by the single author. The description below is intended to make the search reproducible and the provenance of every statement traceable, not to claim systematic-review status. All graphical illustrations were designed by the author using original artwork and conceptual syntheses derived from the cited literature. No previously published figures were reproduced or adapted.

**Sources searched.** The following sources were interrogated between 1 and 30 June 2026, with a coverage and content cutoff of 30 June 2026: PubMed/MEDLINE; ClinicalTrials.gov; the World Health Organization International Clinical Trials Registry Platform (WHO ICTRP), which aggregates the EU Clinical Trials Register/CTIS, ISRCTN, the Chinese Clinical Trial Registry (ChiCTR), and the Japan Registry of Clinical Trials (jRCT); the abstract databases of the American Epilepsy Society (AES 2024 and 2025), the American Academy of Neurology (AAN 2024–2026), and the International Epilepsy Congress (IEC 2025); and the public regulatory records of the US Food and Drug Administration (FDA) and the European Medicines Agency (EMA). Registry searching was performed primarily through ClinicalTrials.gov and WHO ICTRP; the national registers listed above were therefore covered indirectly through ICTRP rather than being searched individually in their native interfaces. This is stated explicitly as a limitation, since ICTRP indexing of ChiCTR and jRCT records is not always complete or contemporaneous, and a small number of regionally registered programmes may consequently have been missed.

**Search concepts.** Bibliographic searching combined disease terms (epilepsy; seizures; developmental and epileptic encephalopathy; Dravet syndrome; Lennox–Gastaut syndrome; drug-resistant epilepsy) with intervention terms (antiseizure medication; anticonvulsant; drug development; pipeline; investigational) and mechanism terms (persistent sodium current; Na_V_; K_V_7/KCNQ; GABA_A_; NMDA; AMPA; metabotropic glutamate; 5-HT_2C_; cholesterol 24-hydroxylase; antisense oligonucleotide; gene therapy; antiepileptogenesis; disease modification), together with free-text searches on each individual compound name and development code. Registry searching used compound names, development codes, and sponsor names filtered to interventional studies in epilepsy. Reference lists of retrieved reviews and of the EILAT XVII progress reports were hand-searched [15,16]. No language restriction was applied at the screening stage, although all included sources are in English.

**Eligibility and prioritization.** Clinical-stage therapies with novel or differentiated mechanisms of action were prioritized, with particular emphasis on phase II/III programmes, genetically targeted therapies, and approaches judged likely to influence clinical practice within the next several years. Approved agents were included selectively where they provide mechanistic reference points (cenobamate, ganaxolone, fenfluramine). Programmes that failed, were discontinued, or were paused were deliberately retained, since their inclusion is essential to an unbiased account of the field. Compounds without any clinical-stage activity were excluded except when cited to illustrate a mechanistic principle. If the same trial appeared in more than one registry or was reported at more than one congress, records were deduplicated by trial registration number, and the most recent and most complete report was used; trial status and phase were confirmed against the registry record on the access date given in the corresponding reference.

**Evidence-provenance framework.** Because a substantial part of the contemporary pipeline has been reported outside the peer-reviewed literature, every substantive statement in this review is assigned to one of seven provenance classes, which are identified in the text, in the tables, and in the reference list: (i) peer-reviewed randomized controlled trial; (ii) peer-reviewed open-label or uncontrolled clinical study; (iii) peer-reviewed preclinical or pharmacological study; (iv) congress presentation or abstract; (v) interim analysis, whether or not congress-reported; (vi) clinical-trial registry record; and (vii) regulatory record or other official public source. Company press releases were not used as primary sources for efficacy data. Findings in classes (iv) to (vii) are introduced with explicitly provisional language (“reported at”, “according to an interim analysis”, “as listed in the trial registry”, “at the time of the literature search”) and are not presented as established results. Efficacy percentages derived from congress presentations or open-label extensions are identified as such at the point of use, in the abstract, in the main text, and in the tables. Readers should assume that any figure so labelled may change on full publication.

**Limitations of the search.** Three limitations follow from the design. Single-reviewer screening introduces selection subjectivity. Congress abstracts are not peer reviewed to the standard of full papers, are frequently updated between meetings, and are sometimes never published in full, so the evidence base for several of the most prominent agents is intrinsically unstable. Finally, a pipeline review is dated by construction: trial status, regulatory milestones, and pipeline counts reported here are current only to 30 June 2026.

## 3. Landscape of the Contemporary ASM Pipeline

A 2024 analysis of publicly disclosed studies identified more than 200 novel epilepsy therapies in preclinical or clinical development; this figure refers to distinct therapeutic programmes, spanning all modalities and all stages from preclinical to registration, as counted by those authors at that date, and is not a stable denominator—an unusually rich pipeline that reflects both the genetic revolution in epilepsy and the commercial attractiveness of orphan-drug designation for rare syndromes [4]. Several structural features distinguish this pipeline from earlier eras.

First, the target landscape, while still anchored in neurotransmitter systems and ion channels, has broadened considerably. GABA_A_ receptors, glutamate receptors, and voltage-gated ion channels (sodium, potassium, calcium) remain the most common targets, but serotonin, cannabinoid, and purinergic receptors are increasingly pursued. In addition, targeting of the mTOR pathway and the ubiquitin-protein ligase *UBE3A* have appeared for specific genetic syndromes. Reestablishing physiological neuronal excitability remains the dominant objective, yet a meaningful minority of drugs now address neuroplasticity, circuit remodelling, neuroinflammation, energy metabolism, and epigenetics—processes implicated in epileptogenesis rather than ictogenesis alone [4,5].

Second, therapeutic modalities are diversifying. Small molecules still represent roughly three quarters of programmes, but antisense oligonucleotides (ASOs), RNA-based therapies, gene therapies, antibodies, and peptides accounted for about a quarter in the 2024 analysis cited—an unprecedented expansion reflecting the rise in genetically defined disease [4].

**Figure 2:**
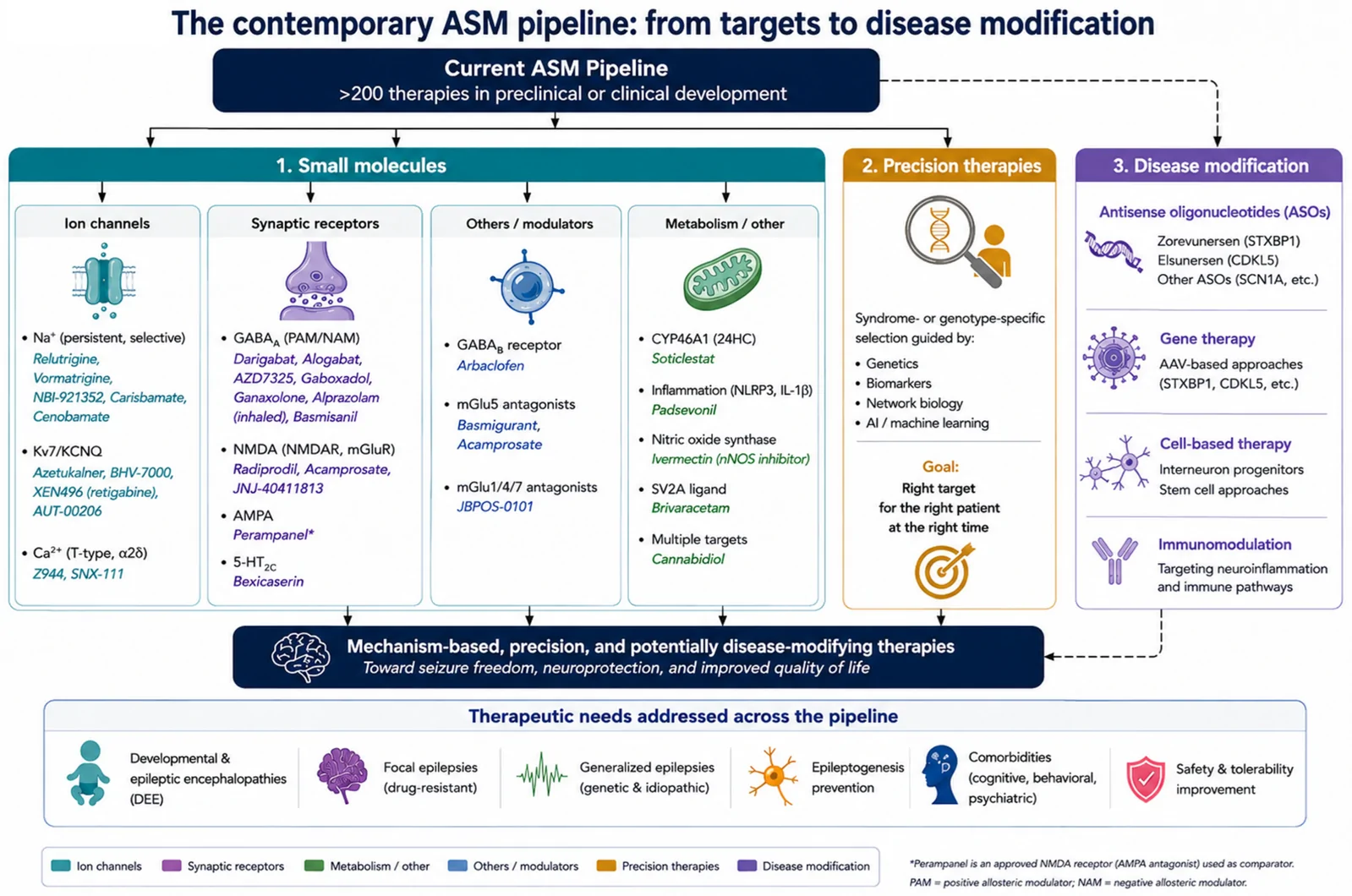
The contemporary antiseizure medication pipeline: From molecular targets to disease modification. Schematic overview of the current antiseizure medication development landscape, organized into three complementary therapeutic domains: mechanism-based small molecules, precision therapies, and disease-modifying approaches. The figure illustrates the convergence of novel pharmacological targets, genomic medicine, biomarker-guided patient stratification, and advanced therapeutic platforms that are collectively reshaping epilepsy treatment toward individualized and potentially disease-modifying care. This schematic is a conceptual synthesis and not a quantitative pipeline census; classification of an approach as disease-modifying denotes therapeutic intent, not demonstrated effect, and development phases shown are current to 30 June 2026. Based on Refs. [1,7,15,17,18,19,20].

**Figure 3:**
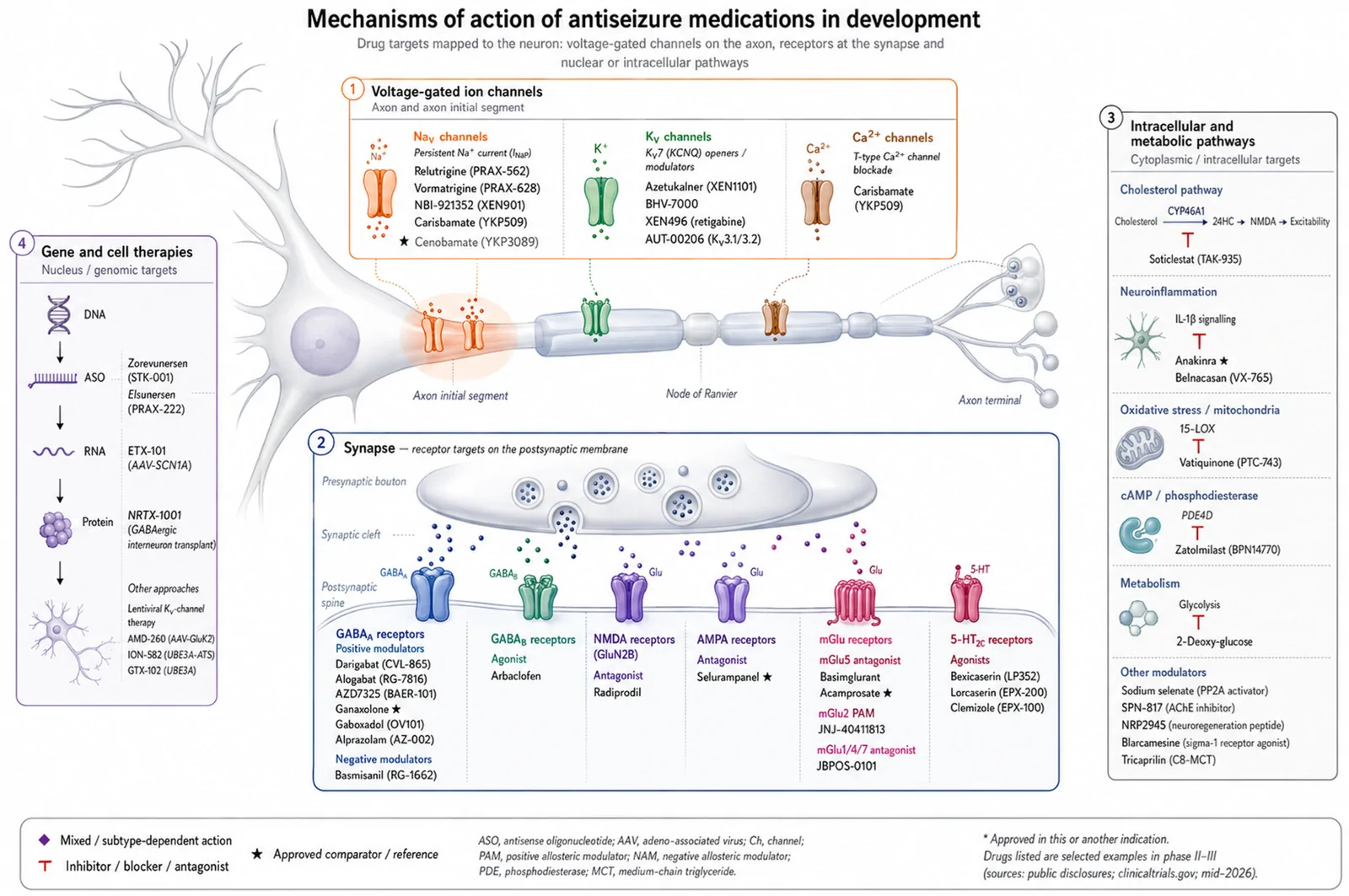
Mechanisms of action of antiseizure medications (ASMs) in development, mapped onto the neuron. Investigational agents are grouped by the anatomical and molecular site at which they act, spanning the axon, the synapse, intracellular/metabolic pathways, and the nucleus. (1) Voltage-gated ion channels (axon and axon initial segment). Sodium-channel agents that preferentially suppress the persistent sodium current (I_NaP): relutrigine (PRAX-562), vormatrigine (PRAX-628), NBI-921352 (XEN901) and carisbamate (YKP509), with cenobamate (YKP3089) shown as an approved reference. Kv7/KCNQ potassium-channel openers/modulators: azetukalner (XEN1101), BHV-7000, XEN496 (retigabine) and AUT-00206 (Kv3.1/3.2). T-type Ca^2+^-channel blockade: carisbamate. (2) Synapse—receptor targets on the postsynaptic membrane. GABA_A-receptor-positive modulators (darigabat [CVL-865], alogabat [RG-7816], AZD7325 [BAER-101], ganaxolone, gaboxadol [OV101], alprazolam [AZ-002]) and the negative allosteric modulator basmisanil (RG-1662); the GABA_B agonist arbaclofen; the GluN2B-selective NMDA-receptor antagonist radiprodil; the AMPA-receptor antagonist selurampanel; metabotropic glutamate-receptor agents (mGlu5 antagonists basimglurant and acamprosate, the mGlu2 positive allosteric modulator JNJ-40411813, and the mGlu1/4/7 antagonist JBPOS-0101); and 5-HT_2_C-receptor agonists (bexicaserin [LP352], lorcaserin [EPX-200], clemizole [EPX-100]). (3) Intracellular and metabolic pathways. The brain-cholesterol pathway (cholesterol → [CYP46A1] 24-hydroxycholesterol [24HC] → NMDA-mediated excitability), inhibited by soticlestat (TAK-935); neuroinflammation (IL-1β signalling; anakinra, belnacasan [VX-765]); oxidative stress/mitochondrial function (15-lipoxygenase [15-LOX]; vatiquinone [PTC-743]); cAMP/phosphodiesterase signalling (PDE4D; zatolmilast [BPN14770]); glycolytic metabolism (2-deoxy-glucose); and other modulators (sodium selenate [PP2A activator], SPN-817 [acetylcholinesterase inhibitor], NRP2945 [neuroregeneration peptide], blarcamesine [sigma-1 receptor agonist], tricaprilin [C8 medium-chain triglyceride]). (4) Gene and cell therapies (nucleus/genomic targets). Along the DNA → RNA → protein axis: the antisense oligonucleotides zorevunersen (STK-001) and elsunersen (PRAX-222); the AAV-*SCN1A* gene therapy ETX-101; and the GABAergic interneuron cell transplant NRTX-1001. Other approaches include lentiviral Kv-channel therapy, AMT-260 (AAV-GluK2), ION-582 (UBE3A-ATS) and GTX-102 (UBE3A). Symbols: ↑ activator, opener or agonist; ⊣ inhibitor, blocker or antagonist; ◆ mixed or subtype-dependent action; ★ approved comparator/reference (approved in this or another indication). Abbreviations: AAV, adeno-associated virus; AChE, acetylcholinesterase; AMPA, α-amino-3-hydroxy-5-methyl-4-isoxazolepropionic acid; ASO, antisense oligonucleotide; cAMP, cyclic adenosine monophosphate; CYP46A1, cholesterol 24-hydroxylase; GABA, γ-aminobutyric acid; GluN2B, NMDA-receptor subunit 2B; I_NaP, persistent sodium current; IL-1β, interleukin-1β; Kv/KCNQ, voltage-gated potassium channel; mGlu, metabotropic glutamate receptor; MCT, medium-chain triglyceride; NaV, voltage-gated sodium channel; NMDA, *N*-methyl-D-aspartate; PAM/NAM, positive/negative allosteric modulator; PDE, phosphodiesterase; PP2A, protein phosphatase 2A; 5-HT_2_C, serotonin 2C receptor; 24HC, 24-hydroxycholesterol. This figure is a conceptual synthesis rather than a presentation of original quantitative data, and the mechanisms shown are the predominant or therapeutically intended actions of each compound rather than exclusive ones; several agents have concentration-dependent secondary effects. The agents shown are selected examples in phase II–III development; targets and compounds were compiled from peer-reviewed publications, congress presentations (American Epilepsy Society 2024–2025; American Academy of Neurology 2026; International Epilepsy Congress 2025) and ClinicalTrials.gov, and are current only to the search cutoff of 30 June 2026.

Third, the centre of gravity has shifted toward the DEEs (Figure 2). Of the compounds identified in advanced (phase II/III) development at the 30 June 2026 search cutoff —more than 40 by the present count, which reflects compounds rather than trials or indications—a clear majority target DEEs: a large, heterogeneous group of more than 250 rare, often single-gene disorders characterized by refractory seizures and abnormal neurodevelopment. Widely recognized examples include Dravet syndrome (DS), Lennox–Gastaut syndrome (LGS), tuberous sclerosis complex (TSC), CDKL5 deficiency disorder, and Angelman, Rett, and fragile X syndromes [5]. The concentration of effort here reflects both the severity of unmet need and the tractability of genotype-matched precision approaches, which are easier to rationalize and, in some cases, to demonstrate in a biomarker-defined population. The sections that follow examine the most clinically advanced and mechanistically distinctive agents within this pipeline, organized by molecular target. These agents are summarized in Table 1 and Figure 3.

## 4. Voltage-Gated Sodium Channels: Functional-State Selectivity and the Persistent Current

Sodium-channel modulation is the oldest and most heavily populated mechanistic class in epilepsy therapeutics, yet it is also the site of its most interesting recent conceptual advance. Classical blockers—phenytoin, carbamazepine, and lamotrigine—act predominantly on the transient sodium current and stabilize the inactivated channel state in a use-dependent but relatively broad manner [5,9]. Their efficacy is real but bounded by tolerability: the channels that drive seizures also sustain normal signalling, so indiscriminate blockade produces dose-limiting dizziness, diplopia, and ataxia, and can paradoxically aggravate seizures in certain gain-of-function channelopathies.

The persistent (or “late”) sodium current, I_NaP_, offers a more selective therapeutic handle. Comprising only about 1–2% of the total sodium current, I_NaP_ fails to inactivate appreciably during sustained depolarization and therefore disproportionately amplifies neuronal responses to synaptic input and supports repetitive and burst firing—the cellular substrates of ictogenesis [9]. I_NaP_ is pathologically elevated in several sodium channelopathies and is implicated in acquired focal epilepsies as well. Part of cenobamate’s high efficacy is attributed to inhibition of the persistent current, which provides indirect clinical support for the target. That support is not decisive: cenobamate is a multi-target agent, and the relative contributions of persistent-current inhibition and of positive allosteric modulation at GABA_A_ receptors cannot be separated in patients [30].

The newest sodium-channel agents are designed around functional-state selectivity: rather than blocking channels tonically, they preferentially engage the hyperexcitable conformations responsible for pathological persistent and repetitive firing while largely sparing normally functioning channels. The intended consequences are a wider therapeutic window, rapid onset without slow titration, and improved tolerability. Two qualifications belong within this framework. First, preference for the persistent current is quantitative, not absolute: in the best-characterized example, PRAX-562 inhibits persistent I_NaP_ with roughly 60-fold preference over tonic block of peak I_Na_—a large but finite ratio, so peak-current block is expected at higher exposures [21]. Second, functional-state selectivity is a statement about channel conformation, not about channel isoform or channel family, and the two are frequently conflated. Isoform selectivity is a separate property, achieved to a striking degree by NBI-921352, which inhibits Na_V_1.6 with selectivity ratios of 134- to 756-fold over other neuronal Na_V_ isoforms while itself acting through preferential binding of the inactivated state [27]. Neither property implies the absence of secondary actions on other channel families, and for most agents in this class such actions have not been systematically excluded in the public literature [5]. The principal molecular targets discussed throughout this review, and the agents acting on each, are summarized in Figure 3.

### 4.1. Relutrigine (PRAX-562)

Relutrigine is a once-daily oral small molecule engineered as a preferential inhibitor of the persistent sodium current, with preference for the disease-state channel. In peer-reviewed preclinical work it inhibited ATX-II-evoked and Na_V_1.6-N1768D-mediated persistent I_Na_ with half-maximal inhibitory concentrations of 141 and 75 nmol/L respectively, an approximately 60-fold preference over tonic block, and a protective index of about 16 in the maximal electroshock model compared with roughly 5.5 for carbamazepine and lamotrigine [17,21,22]. In *SCN2A*, *SCN8A*, and other DEE mouse models it produced dose-dependent seizure inhibition; in some models, to complete control; and it was generally well tolerated across three phase I studies, with biomarker changes consistent with sodium-channel engagement [22].

That population—*SCN2A*- and *SCN8A*-related DEEs—comprises severe, early-onset encephalopathies caused by pathogenic variants in genes encoding sodium-channel α-subunits. These conditions feature frequent treatment-refractory seizures, profound developmental impairment, and high mortality, and crucially have no approved targeted therapy [17]. Gain-of-function variants increase persistent sodium current, providing a direct mechanistic rationale for a state-selective I_NaP_ inhibitor [9].

The phase II EMBOLD study, a randomized, double-blind, placebo-controlled trial with open-label extension (OLE), enrolled children aged 2–18 years with genetically confirmed disease and at least eight countable motor seizures over four weeks. In cohort 1 (*n* = 16; 7 *SCN2A*, 9 *SCN8A*)—an exceptionally severe population that had failed roughly three prior treatments on average—investigators reported a placebo-adjusted reduction in monthly motor seizures of approximately 46%, with more than 30% of patients achieving seizure freedom on the drug, alongside caregiver- and clinician-reported gains in alertness, communication, behaviour, and seizure severity [23]. These are preliminary findings presented at the 2024 and 2025 AES Annual Meetings which, at the time of the literature search, have not yet appeared as a full peer-reviewed publication. In the open-label extension the median seizure reduction reportedly reached approximately 75%; five patients experienced seizure-free intervals exceeding 28 days, and one beyond 200 days, and by month 11 the average reduction was reported as approximately 90%, with more than two months between seizures on average. Open-label extension figures of this kind reflect a self-selected population of tolerators and responders and are subject to enrichment, attrition, and ascertainment bias; they are not comparable with placebo-controlled estimates [22,23]. According to an interim analysis reported at the 2025 AES Annual Meeting, the registrational cohort 2 (*n* = 53) was stopped early in November 2025 on the recommendation of the independent Data Monitoring Committee for overwhelming efficacy, with a placebo-adjusted reduction of approximately 53%. Two cautions apply: this is an interim analysis awaiting full peer-reviewed publication, and effect estimates from trials stopped early for benefit may be prone to overestimation [17,25]. Relutrigine was generally well tolerated, with no drug-related serious adverse events and no required dose reductions [17].

Relutrigine holds FDA Orphan Drug and Rare Pediatric Disease designations (for *SCN2A*-DEE, *SCN8A*-DEE, and DS), FDA Breakthrough Therapy Designation for *SCN2A*/*SCN8A*-DEE, and EMA Orphan Drug and Breakthrough designations [22,24]. According to the FDA regulatory record consulted on 30 June 2026, an NDA was submitted in early 2026 and accepted with Priority Review, carrying a PDUFA target action date of 27 September 2026; an anticipated action date is not an approval, and the review outcome was unknown at the time of writing. If approved, relutrigine would be the first targeted therapy for these conditions [25]. Because sodium-channel blockers are used broadly across DEEs, the EMERALD study is evaluating relutrigine in DEEs irrespective of etiology, with completion anticipated in the second half of 2026 [24].

### 4.2. Vormatrigine (PRAX-628)

Vormatrigine is a functionally selective oral small molecule targeting the hyperexcitable sodium-channel state, administered once daily for adult focal-onset seizures (FOS) and generalized epilepsy. Sponsor-reported in vitro data indicate preferential activity at the hyperexcitable channel state; in vivo the compound showed high potency in the maximal electroshock (MES) model, an assay with good predictive value for generalized tonic–clonic and focal seizures but with well-recognized limitations as a model of pharmacoresistance. These characterizations have been presented at congresses and in company disclosures rather than in a full peer-reviewed pharmacology paper, and the compound’s activity at other ion-channel families has not been reported publicly. A first-in-human study in 40 healthy volunteers showed it could be dosed to more than 15-fold above the predicted human equivalent of the rodent MES EC_50_ with good tolerability and no clinically significant ECG, vital-sign, or neurological findings—evidence of an unusually wide projected therapeutic window [26].

In the open-label phase II RADIANT study, conducted in heavily pretreated patients with FOS or generalized epilepsy taking one to three concomitant ASMs, investigators reported that vormatrigine 30 mg/day produced a 56.3% median reduction in seizure frequency over eight weeks, with 54% of patients achieving at least a 50% response within the first week and 22% reaching complete seizure reduction over the final 28 days; in a generalized/photosensitive arm, the 45 mg dose abolished generalized photoparoxysmal events in all treated patients [26]. These are preliminary findings presented at the 2025 AES Annual Meeting and the 36th International Epilepsy Congress. As an uncontrolled study, these open-label figures cannot be compared directly with placebo-controlled results, and expectations are often frustrated in double-blind studies (see Section 13 and Section Critical Appraisal of Contemporary Antiseizure-Drug Development).

The controlled experience proved more sobering. POWER1, a double-blind, randomized phase II/III trial in adults with FOS (vormatrigine 20 mg/day for six weeks then 30 mg/day for six weeks versus placebo over 12 weeks; primary endpoint, percent change in monthly seizure frequency), did not meet its primary endpoint in this highly refractory population, as reported in June 2026. The 50% responder-rate secondary endpoint was met, seizure reduction on the 30 mg dose was more pronounced during the second half of the study, the drug was generally well tolerated (adverse-event discontinuations below 10%), and approximately 90% of treated patients elected to continue into the OLE; enrolment in the companion POWER2 study was paused while the study is reassessed [18]. The contrast between RADIANT and POWER1 is discussed in Section 13, as it exemplifies a recurring pattern in ASM development.

### 4.3. Elsunersen (PRAX-222) and the Integrated Sodium-Channel Strategy

Elsunersen is an ASO designed to selectively reduce *SCN2A* expression in patients with gain-of-function *SCN2A*-DEE, addressing the molecular cause rather than the downstream current, with clinical data anticipated in 2026 [24]. Together, relutrigine (state-selective small-molecule I_NaP_ inhibition), vormatrigine (functional-state modulation for common epilepsy), and elsunersen (genetic downregulation of a gain-of-function channel) constitute a mechanism-based strategy addressing sodium-channel-driven hyperexcitability across the disease-severity spectrum.

### 4.4. Other Sodium-Channel Approaches

NBI-921352 (XEN901) is a first-in-class selective inhibitor of Na_V_1.6 (encoded by *SCN8A*), in phase II for both *SCN8A*-DEE and adult FOS—an isoform-specific expression of the “right channel, right patient” reasoning. Its preclinical pharmacology is unusually well documented for an agent at this stage, including isoform-selectivity ratios, state dependence, and preservation of firing in fast-spiking interneurons in which Na_V_1.1 predominates; controlled clinical efficacy data have not yet been published [5,27]. Carisbamate (YKP509), a mono-carbamate related to felbamate and cenobamate, reduces repetitive firing through voltage-gated sodium-channel inhibition, with additional modulation of T-type calcium channels; after inconsistent earlier results in focal epilepsy, it is being evaluated in a global phase III trial in LGS, with reduction in drop seizures as the primary objective [5,15].

## 5. Potassium Channels: K_V_7 Openers and Related Modulators

Opening neuronal K_V_7 (KCNQ) potassium channels stabilizes the resting membrane and reduces repetitive firing through a mechanism distinct from nearly all marketed ASMs. The concept was clinically established, though commercially curtailed, by retigabine (ezogabine), which was withdrawn over retinal and dermatological toxicity. Retigabine is also a reminder that a “channel opener” label can conceal a broader pharmacology: besides opening K_V_7.2–7.5 channels, it directly potentiates GABA_A_ receptor currents—with a preference for extrasynaptic δ-subunit-containing receptors—and increases GABA synthesis, ancillary mechanisms that plausibly contributed to its clinical spectrum [5,12,13].

Azetukalner (XEN1101) is a second-generation, K_V_7.2/7.3-preferring opener with an approximately 10-day half-life that permits once-daily dosing with food and no formal titration. The word “selective” requires further insights. Reported selectivity is subtype-relative rather than absolute: in heterologous expression assays azetukalner is approximately fourfold more potent at K_V_7.2/7.3 than at K_V_7.3/7.5 or K_V_7.4, with screening selectivity over unrelated channels and receptors reported as greater than 100-fold, including absence of the GABA_A_ activity seen with retigabine. Selectivity of this order is a genuine pharmacological advance, but it is measured in vitro at defined concentrations and does not establish exclusivity in vivo. K_V_7.4 and K_V_7.5 channels are abundant in vascular and detrusor smooth muscle, so systemic actions are anticipated for any K_V_7-directed agent, and no independent characterization of azetukalner’s secondary pharmacology at therapeutic brain concentrations is publicly available [14]. The proposition that azetukalner may not be specific for the opening of M-type K^+^ currents alone is therefore reasonable, and the wording used throughout this review reflects it [15]. In the phase IIb X-TOLE study it produced robust, dose-dependent reductions in focal seizure frequency in a heavily pretreated population, with rapid onset [32]. The phase III X-TOLE2 trial, evaluating 15 mg and 25 mg doses against placebo in adults with refractory FOS, was reported as positive at the 2026 AAN Annual Meeting, with median reductions in monthly focal-seizure frequency of approximately 53% and 35% for the 25 mg and 15 mg doses versus about 10% for placebo, among the highest placebo-adjusted efficacies reported in a pivotal FOS studies, notable given that a substantial proportion of participants were taking or had discontinued cenobamate [7]. An interim analysis of the X-TOLE open-label extension presented at the 2026 AAN Annual Meeting reported progressive improvement, with median reductions reaching roughly 90% by month 48. A figure of this magnitude from an open-label extension is not a measure of drug effect: patients still enrolled at four years are by construction those who tolerated and responded to the drug, and the estimate is subject to enrichment, attrition, and ascertainment bias as well as to changes in concomitant medication [33]. Azetukalner is also in phase III for primary generalized epilepsy and is being studied in major depressive disorder. If approved, it would be the first K_V_7 opener available for epilepsy [7].

Related developments include BHV-7000 (BNP-25203), a K_V_7.2/7.3 modulator in phase II/III for both *KCNQ2*-DEE (precision use) and focal epilepsy; XEN496, a pediatric retigabine formulation repurposed as precision therapy for *KCNQ2*-DEE; and AUT-00206, a K_V_3.1/3.2 modulator in phase II for fragile X syndrome that enhances cortical network synchronization consistent with restored parvalbumin-interneuron function [5].

## 6. GABA_a_ Receptor Modulation: Subtype-Selective “GABAkines” and Neurosteroids

GABA_A_ receptor enhancement is a venerable strategy, but the current generation aims for subtype selectivity to dissociate antiseizure efficacy from the sedation, tolerance, and dependence of classical benzodiazepines. Because the sedative effect of benzodiazepines is mediated chiefly by α_1_-containing receptors, whereas α_2_/α_3_ receptors mediate anxiolysis and antiseizure effects, α_1_-sparing positive allosteric modulators (PAMs)—“GABAkines”—are designed to retain benefit with fewer liabilities. As with the channel modulators above, subtype “selectivity” in this class denotes relative efficacy or affinity across α-subunit-containing receptor populations rather than exclusive occupancy of one subtype. The physiological consequence also depends on regional receptor composition, synaptic versus extrasynaptic localization, and network context, so a subtype label does not by itself predict the clinical profile [5,8].

The most advanced example is darigabat (CVL-865), an α_2/3/5_-selective PAM that also acts as a partial agonist at the benzodiazepine site, properties expected to minimize tolerance and dependence. Darigabat showed antiseizure activity in the kainate mesial temporal lobe model of drug-resistant focal epilepsy and proof-of-concept activity in the photosensitivity paradigm—a pharmacodynamic signal of cortical target engagement rather than a demonstration of clinical antiseizure efficacy—and it advanced into phase II proof-of-concept trials in drug-resistant focal seizures; its developer was subsequently acquired by AbbVie, which has continued the programme [34]. Alogabat is an α_5_-selective PAM developed for Angelman syndrome, where partial loss of α_5_-subunit-coding genes provides a precision rationale; AZD7325 (BAER-101), an α_2_/α_3_-selective PAM, is in phase II for fragile X syndrome; and gaboxadol (OV101), a high-affinity agonist at extrasynaptic δ-subunit-containing receptors that mediate tonic inhibition, is in development for fragile X syndrome [5].

Neuroactive steroids constitute a distinct GABA_A_ approach, acting at both synaptic and extrasynaptic receptors. Ganaxolone, an analogue of allopregnanolone, is already approved for seizures associated with CDKL5 deficiency and is in phase II/III as an intravenous therapy for refractory status epilepticus (SE)—a rational target because SE becomes benzodiazepine-resistant partly through internalization of synaptic GABA_A_ receptors, whereas extrasynaptic receptors, accessible to neurosteroids, persist in the membrane [35]. Other GABA-directed programmes include the negative allosteric modulator basmisanil for Angelman and Dup15q syndromes, the GABA_B_ agonist arbaclofen for fragile X syndrome, and an inhaled formulation of alprazolam (AZ-002) in phase III for rapid termination of cluster seizures—conceptually distinct from existing nasal, buccal, and rectal benzodiazepine rescue routes [5].

## 7. Glutamate Receptor Modulation

Six advanced-development drugs target ionotropic (NMDA, AMPA) or metabotropic glutamate receptors, acting as positive allosteric modulators (PAMs), negative allosteric modulators (NAMs), or competitive antagonists depending on the target. These descriptors are not interchangeable and carry different implications for use-dependence, ceiling effects, and off-target liability; each is used here as reported by the primary source for the compound concerned. Glutamatergic agents are also unusually susceptible to network-level effects that receptor pharmacology alone does not predict, since the same receptor subtypes are expressed on excitatory and inhibitory neurons alike [5]. To date, only one glutamatergic antagonist—perampanel, an AMPA receptor antagonist—has been approved, and its efficacy is not superior to existing ASMs, underscoring both the appeal and the difficulty of this target class [4].

The most precision-oriented agent is radiprodil, a NAM selective for NR2B (GluN2B)-containing NMDA receptors, developed for seizures and behavioural manifestations associated with gain-of-function variants in *GRIN1/2A/2B/2D*—genetically defined encephalopathies in which excess NMDA-receptor function is mechanistically central. Among metabotropic approaches, basimglurant and acamprosate target mGlu5 (the former in phase II for TSC and fragile X syndrome, the latter in phase III for fragile X syndrome); JNJ-40411813 (ADX71149), an mGlu2 PAM, is in phase II for focal epilepsy with suboptimal response to levetiracetam or brivaracetam, having shown synergy with levetiracetam in the 6 Hz model; and JBPOS-0101, an mGlu1/4/7 antagonist, is in phase II for DEEs and refractory SE. Selurampanel, an AMPA-receptor antagonist, remains in earlier evaluation [5].

## 8. Serotonergic Mechanisms

The unexpected, substantial efficacy of fenfluramine in DS—mediated through serotonergic signalling—catalyzed a wave of development of 5-HT receptor drugs, several seeking to retain efficacy while avoiding the 5-HT_2B-_mediated cardiac valvulopathy and pulmonary hypertension associated with non-selective agents [5,41].

Bexicaserin (LP352), a high-efficacy (developer-designated “superagonist”) and subtype-preferring 5-HT_2C_ agonist, is the most advanced. Selectivity over 5-HT_2B_ is the pharmacological objective of this class, since 5-HT_2B_ agonism underlies the valvulopathy and pulmonary hypertension associated with earlier non-selective agents; as elsewhere, selectivity is relative, and whether it is clinically sufficient will be established only by prolonged cardiac surveillance. In the phase Ib/2a PACIFIC trial in patients with DEEs aged 12–65, bexicaserin reduced countable motor seizures by a median of approximately 60% versus roughly 17% with placebo, with the greatest effect in DS (about 75% reduction, though only 3 Dravet patients were enrolled) and a 60% responder rate. This is peer-reviewed randomized evidence, but from a small phase Ib/2a study; the syndrome-level subgroup estimates rest on few patients and are hypothesis-generating pending the phase III DEEp programme [37]. The open-label extension reportedly showed durable reductions (around 59% at 52 weeks, sustained to roughly 54–60% at 18–24 months) with a favourable tolerability profile and high retention—findings presented at the 2025 AES Annual Meeting and subject to biases inherent in open-label studies—and preclinical data suggested reductions in seizure-associated respiratory arrest in a SUDEP model [38]. PACIFIC was published in 2026, and following acquisition of the originator by Lundbeck, bexicaserin advanced into the global phase III DEEp study (including DEEp SEA in DS and DEEp OCEAN in broad DEEs); it holds FDA Breakthrough Therapy Designation for DEE-associated seizures, Orphan Drug designations for DS and LGS, and Rare Pediatric Disease designation for DS [37,38]. Related 5-HT_2C_ agonists (lorcaserin/EPX-200, clemizole/EPX-100) and the 5-HT_2A_ agonist psilocybin (NM-1001) are in earlier development; whether any will match fenfluramine’s efficacy in DS remains to be determined [5].

## 9. Novel and Orthogonal Small-Molecule Mechanisms

Soticlestat (TAK-935) inhibits cholesterol-24-hydroxylase (CYP46A1), a brain-specific enzyme that converts cholesterol to 24S-hydroxycholesterol (24HC), a positive allosteric modulator of NMDA receptors. By lowering 24HC, soticlestat is proposed to reduce NMDA-mediated hyperexcitability, with possible secondary effects on glutamate clearance and neuroinflammation [39]. After encouraging phase II data (ELEKTRA), the phase III SKYLINE (DS) and SKYWAY (LGS) studies narrowly missed their primary endpoints while showing clinically meaningful effects on key secondary measures—an outcome that tempered but did not extinguish interest in the mechanism [39,40].

Cenobamate, although approved, merits inclusion as a recent validation of multimodal pharmacology: it combines preferential inhibition of the persistent sodium current with positive allosteric modulation of GABA_A_ receptors at a non-benzodiazepine site [30]. This dual action is widely credited for its high efficacy—maintenance-phase seizure-freedom rates unmatched among focal-epilepsy pivotal trials of the past quarter-century [28,29], and the highest probability of seizure freedom among approved and investigational agents in a 2026 network meta-analysis. Network meta-analyses of adjunctive ASM trials rest on indirect comparisons across studies differing in population severity, background therapy, era, and placebo response; their rankings are informative but are not a substitute for head-to-head trials [19,31]. Early recognition of a DRESS risk led to a slow-titration protocol that effectively mitigated it [30]. Its persistent-current component supports a broader argument that multi-target agents may outperform single-target blockers.

A diverse set of additional mechanisms is represented in advanced development: vatiquinone (PTC-743), a 15-lipoxygenase inhibitor for mitochondrial epilepsy; sodium selenate, a protein-phosphatase-2A activator being investigated on the basis of a disease-modification hypothesis in temporal lobe epilepsy, for which clinical disease-modifying efficacy has not been demonstrated; 2-deoxy-glucose, a glycolytic inhibitor; the sigma-1 receptor agonist blarcamesine (Rett and related syndromes); the PDE4D inhibitor zatolmilast (BPN14770) for fragile X syndrome; SPN-817, a synthetic huperzine A enhancing cholinergic and GABAergic signalling; and NRP2945, a neural-regeneration peptide that upregulates GABA_A_ subunit expression. The read-through agent ataluren, which promotes suppression of premature stop codons, was tested in nonsense-variant DS and CDKL5 deficiency but did not show convincing efficacy in a small phase II trial—an illustration that mechanistic plausibility does not guarantee clinical benefit [5].

## 10. Anti-Inflammatory, Metabolic, and Microbiome-Based Strategies

Recognition of neuroinflammation and metabolic dysfunction in epileptogenesis has produced a cluster of studies targeting non-neuronal pathways. Anakinra (IL-1 receptor antagonist) and the caspase-1/IL-1β inhibitor belnacasan (VX-765) target inflammatory cascades, with anakinra used anecdotally in febrile infection-related epilepsy syndrome (FIRES). Ketogenic and metabolic approaches include tricaprilin, a medium-chain-triglyceride (C8) agent for infantile spasms. Reflecting growing interest in the gut–brain axis, microbiome-directed strategies—*Lactobacillus* probiotics for refractory childhood epilepsy and fecal microbiota suspension—have entered phase II and II/III evaluation, respectively. These approaches remain early in development, rest largely on preclinical rationale and small uncontrolled series, and their clinical value is not established, but they substantially broaden the conceptual scope of antiseizure therapy [5].

## 11. Gene-Directed and Molecular Therapies

Perhaps the most transformative frontier is the application of genetic medicine to monogenic epilepsies. Several modalities have entered clinical efficacy testing (Table 2). The most advanced is zorevunersen (STK-001), an ASO that uses targeted augmentation of nuclear gene output (TANGO) to increase productive *SCN1A* transcription and restore Na_V_1.1 expression in DS, most cases of which result from *SCN1A* haploinsufficiency [42]. The open-label phase I/IIa MONARCH study and its extensions, now published in full, reported substantial and durable reductions in convulsive seizure frequency together with gains in cognition and behaviour. It is worth being precise about what this does and does not establish. The evidence is peer-reviewed but uncontrolled: the observed seizure and developmental outcomes are consistent with a disease-modifying effect, and they are equally consistent with symptomatic benefit superimposed on the developmental trajectory expected in treated Dravet syndrome, with regression to the mean, and with caregiver expectation in an unblinded setting. Separating these interpretations will require the sham-controlled EMPEROR trial and, ultimately, evidence that such benefits persist or natural history is altered [20]. Zorevunersen received FDA Breakthrough Therapy Designation and is now in the global, sham-controlled phase III EMPEROR study (developed with Biogen), enrolling approximately 150 children with non-gain-of-function *SCN1A* variants; enrolment completion was expected in 2026 with a data readout anticipated in mid-2027 and a rolling NDA submission to follow [20,43].

Other genetic drugs include ETX-101, a non-replicating AAV vector that upregulates *SCN1A* and increases Na_V_1.1 density in GABAergic interneurons for *SCN1A*-DS (phase I/II); NRTX-1001, a GABA-releasing regenerative neural cell therapy for unilateral mesial temporal lobe epilepsy with hippocampal sclerosis (phase I/II); a lentiviral gene therapy delivering an engineered potassium channel for refractory focal epilepsy; and AMT-260, a gene therapy reducing kainate-receptor GluK2 subunit expression. In Angelman syndrome, the ASOs ION-582 and GTX-102 aim to inhibit the *UBE3A* antisense transcript and reactivate the silenced paternal allele. Alongside elsunersen, these developments signal a shift toward interventions that target underlying genetic causes and that may, if the hypothesis holds, offer durable benefit. All these programmes are nonetheless at the first-in-human or early interim stage; the available outcomes are predominantly safety, biomarker, and uncontrolled seizure-frequency data, and none have yet demonstrated clinical disease modification. Questions of durability, long-term safety, immunogenicity, delivery, redosing, and cost-effectiveness remain entirely open [5].

## 12. From Seizure Suppression to Disease Prevention and Modification

A further paradigm shift extends the field’s ambition beyond suppressing established seizures toward preventing or modifying epileptogenesis itself. Approximately 20% of epilepsies follow acute CNS insults—traumatic brain injury, stroke, and infection—in which patients present at the time of injury but seizures emerge only after a latent period of weeks to years [44]. This latency constitutes a window of opportunity, yet no preventive or disease-modifying treatment is approved [45]. Three objectives are frequently conflated and are worth separating. *Seizure prevention* denotes suppression of seizure occurrence in a patient at risk; *epilepsy prevention* (*antiepileptogenesis*) denotes preventing development of the enduring predisposition itself, so that epilepsy does not arise; and *disease modification* denotes altering the trajectory of established disease—its severity, comorbidity burden, or natural history—in a way that persists after the intervention is withdrawn. A treatment that delays seizure onset while it is being given has not thereby prevented epilepsy, and a treatment that reduces seizures has not thereby modified disease. An early clinical proof of principle for the second objective does exist: in the EPISTOP trial in TSC, initiating vigabatrin at the onset of electrophysiological change but before clinical seizures prolonged the time to first seizure and reduced the incidence of clinical and drug-resistant epilepsy compared with standard care. This is an important result; however, it is a single trial, only partly randomized and in a small genetically defined population with a strongly predictive electrographic biomarker; its generalization to acquired epileptogenesis remains unproven [46]. Ongoing trials are evaluating perampanel and eslicarbazepine acetate for prevention of post-stroke epilepsy, and several studies suggest an antiepileptogenic effect of statins after stroke [45]. More than 20 interventions have prevented or modified acquired epilepsy in animal models, and multi-laboratory consortia (notably EpiBioS4Rx and TAPTE) are developing standardized, cross-validated post-traumatic epilepsy models to enable rigorous therapeutic testing [4]. Some preclinical data further suggest that disease progression, or “secondary epileptogenesis,” can be modified even after epilepsy onset [5]. If substantiated, prevention or modification of epilepsy—long described as the field’s “Holy Grail”—would represent the most consequential advance of all. The evidentiary bar should be set accordingly. Preclinical rationale, or a reduction in seizure frequency during treatment, does not justify the label. Demonstrating disease modification requires evidence of altered natural history: benefit sustained after treatment withdrawal, a reduction in the incidence of epilepsy rather than a delay in its onset, or improvement in developmental trajectory not wholly attributable to seizure reduction—with follow-up long enough to distinguish these outcomes and with prespecified definitions. No agent in the current pipeline has yet met such standards [45].

## 13. The Translational Gap: Open-Label Promise Versus Randomized Reality

The contrasting trajectories of relutrigine and vormatrigine crystallize a lesson that recurs throughout ASM development. Relutrigine’s randomized, placebo-controlled EMBOLD data—reinforced by an interim analysis stopped early for efficacy—translated cleanly into a registrational package. Vormatrigine’s large open-label RADIANT effect did not fully reproduce in the blinded POWER1 trial, where the primary endpoint was missed despite a preserved responder signal and a dose-dependent trend [18,26].

**Figure 4:**
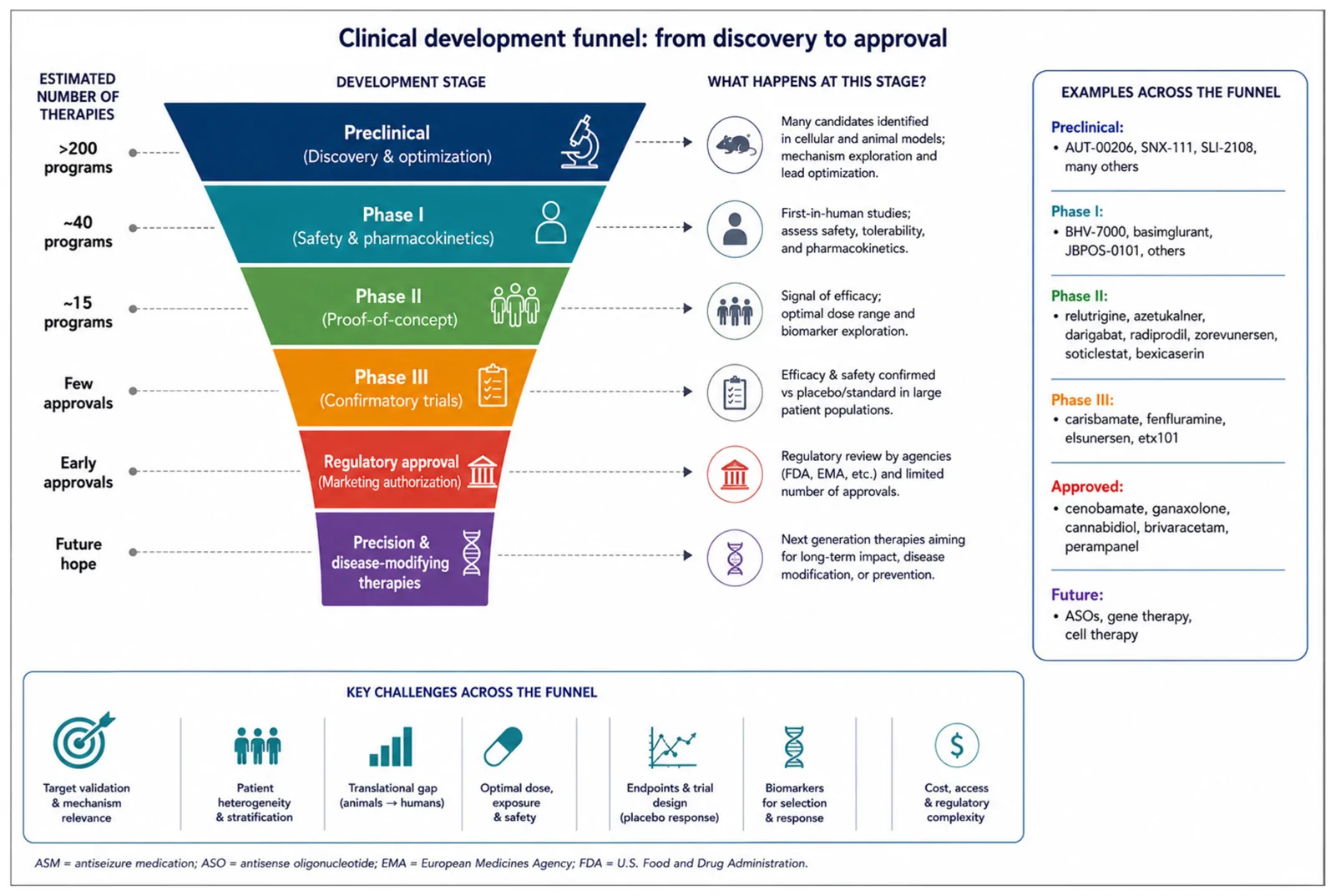
Clinical development funnel for antiseizure medications: From discovery to approval. Overview of the clinical development pathway for novel antiseizure therapies, from preclinical discovery through phase I–III clinical trials and regulatory approval. The figure highlights the progressive attrition of candidate compounds across development stages, the principal objectives of each phase, representative investigational therapies, and key scientific and regulatory challenges that influence successful translation into clinical practice. The funnel is illustrative: the numbers of programmes depicted at each stage are schematic and are not a count of active epilepsy programmes at any given date. Compounds shown are examples current to 30 June 2026.

This pattern is neither idiosyncratic nor new [47]. CNS drugs exhibit higher attrition than non-CNS drugs, driven principally by failure to demonstrate efficacy in large randomized trials, and more than half of agents with positive phase II results fail in phase III—the progression from phase II to phase III being the most consequential decision in drug development (Figure 4) Recent epilepsy failures are instructive (Table 3). Padsevonil, the first rationally designed multimodal ASM (combining synaptic-vesicle protein modulation with benzodiazepine-site GABA_A_ activity), showed robust efficacy across pharmacoresistant animal models and a positive phase IIa proof-of-concept, yet had only modest effect and failed to separate from placebo on primary endpoints in larger phase IIb and III trials [48]. Brexanolone (allopregnanolone), biologically plausible for super-refractory SE, succeeded in open-label evaluation but failed a placebo-controlled phase III trial; bumetanide, proposed for neonatal seizures on the basis of NKCC1 inhibition, founded on a flawed premise, inadequate brain target engagement, and ototoxicity; and ivermectin, a non-selective GABA_A_ PAM with poor blood–brain-barrier penetration, produced only modest effects at near-toxic doses before development was halted [5].

**Figure 5:**
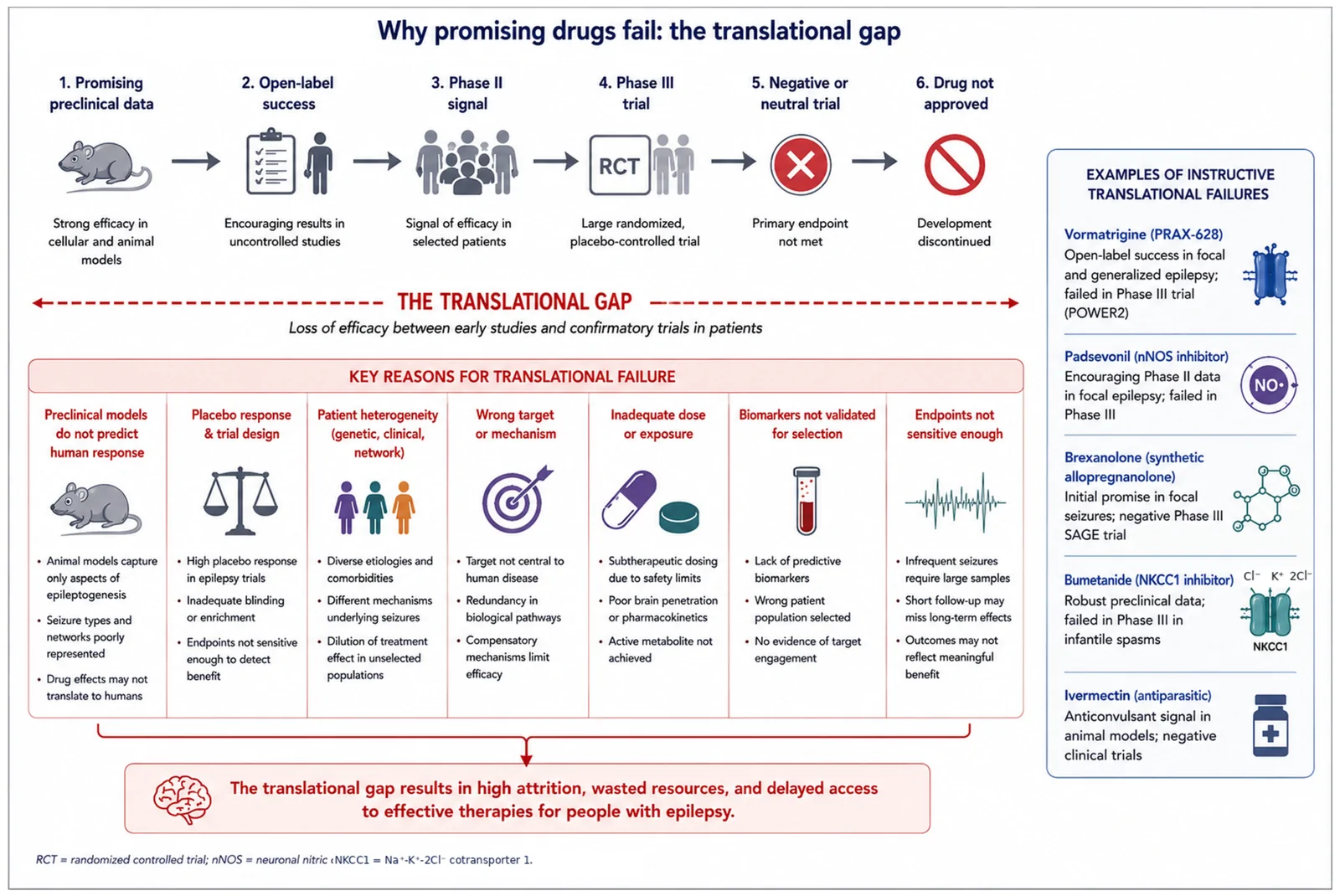
The translational gap in antiseizure drug development. Conceptual framework illustrating the frequent loss of efficacy observed between encouraging preclinical findings and negative or inconclusive late-stage clinical trials. Major contributors include limitations of experimental models, placebo response, patient heterogeneity, inadequate target selection, suboptimal pharmacokinetics or dosing, insufficient biomarker validation, and insensitive clinical endpoints. Representative examples of unsuccessful development programmes illustrate the challenges that continue to impede translation from experimental discoveries to effective clinical therapies. Inclusion of a programme among these examples indicates that a trial did not meet its primary endpoint or was discontinued; it does not establish that the underlying target is invalid (see Section Critical Appraisal of Contemporary Antiseizure-Drug Development).

The common thread is that animal-model efficacy and uncontrolled clinical signals, however striking, are necessary but not enough. It is equally important not to overread the failures. A negative phase III trial does not by itself invalidate a target or even a drug; it may reflect the molecule, the dose, the population, the endpoint, or the design (see Section Critical Appraisal of Contemporary Antiseizure-Drug Development). The placebo response, regression to the mean, and the particular difficulty of demonstrating benefit in heavily pretreated, refractory populations all conspire against single-arm extrapolation [49]. For functionally novel agents in common epilepsy, this argues for careful dose optimization and adequately powered, blinded designs before efficacy is considered established. It also helps explain why the rare-disease, biomarker-anchored, large-effect-size setting (as in *SCN2A*/*SCN8A*-DEE or DS) has recently proven a more tractable path to registration than the crowded, modest-effect world of adjunctive adult focal epilepsy (Figure 5).

### Critical Appraisal of Contemporary Antiseizure-Drug Development

The preceding sections describe what is already being developed. This section sets out how, in my assessment as a practicing epileptologist engaged in epilepsy clinical research and trial conduct, such evidence supporting that development should be read—and where the methodology of contemporary antiseizure-drug development could be improved. My analysis is offered constructively, intending to help set a path forward for future drug development and, ultimately, reaching meaningfully clinical outcomes for patients and caregivers.

**Open-label inflation.** The single most consistent distortion in this field is the uncritical contrast of open-label and placebo-controlled results. Reductions of 75–90% are reported from relutrigine and azetukalner extensions, roughly 59% from the bexicaserin extension, and 56% from the uncontrolled RADIANT study of vormatrigine; the corresponding placebo-adjusted estimates, where they exist, are 46–53%, about 40 percentage points over placebo for azetukalner, and according to publicly reported, not yet peer-reviewed results, the blinded POWER1 trial did not meet its primary endpoint, although it met its secondary endpoint and over 90% of patients are reported to have transitioned to open-label. This gap is not a mirage; it is the arithmetic effect of the unblinding nature of open-label observation. Absence of blinding builds expectation effects in caregivers and investigators alike; enrolment typically follows a high-seizure qualifying period, so regression to the mean operates from the first day of treatment; extensions mainly retain responders and tolerators and undercount everyone else, so the denominator improves as the study proceeds, unlike intention-to-treat trials; concomitant medications are also adjusted and added; and the diligence of seizure counting itself changes once families believe the drug is working. The practical implications are straightforward and understandable. Open-label extension results should be reported against the full enrolled cohort as well as the completer population, with explicit attrition accounting; they should never be placed in the same sentence, table, or slide as a placebo-adjusted figure without an explicit statement that the two may not be comparable; and reviewers, guideline committees, and clinicians should treat large open-label results as a reason to run a controlled trial rather than as a partial substitute for one, even if the temptation for considering in new therapies is overwhelming.

**Placebo response and its instability.** Placebo responder rates in adjunctive epilepsy trials have risen over recent decades and vary substantially by region, trial size, and baseline seizure frequency; much of what is called a placebo effect is in fact just natural variability in seizure counts combined with regression to the mean [50,51,52]. In DEE populations the problem is amplified: cohorts are small, seizure frequency is highly variable within and between patients, seizure precipitants may affect one part of a trial more than another (i.e., pulmonary or viral infections during winter and autumn), outcomes depend on caregiver diaries, the accuracy of which is itself influenced by expectation, and a handful of participants at a single site could move a median. Trials in these syndromes should therefore use prospective baselines long enough to stabilize the estimate, prespecify handling of extreme values, stratify by region and by baseline frequency, and—when feasible—validate diary-based counts with objective seizure detection. In a few diseases this is more feasible than others. For instance, in many DEEs, in which patients experience a high number of seizures (sometimes daily), even shorter Video-EEG recordings could serve as proof-of-concept to validate caregiver’s seizure counts and even evaluate efficacy, although I acknowledge that this is mainly exploratory.

**Patient heterogeneity: Genotype is not mechanism.** Precision development in DEEs rests on an assumption that deserves more scrutiny than it usually receives, namely that a shared gene implies a shared mechanism. Unfortunately, it frequently does not. Within a single gene, gain-of-function and loss-of-function variants may predict opposite responses to the same drug—the paradigm case being sodium-channel blockade, which benefits early-onset gain-of-function *SCN1A*-related disease and may aggravate later-onset loss-of-function disease (i.e., Dravet Syndrome). *GRIN*-related disorders span the same functional spectrum, which is why radiprodil is being developed specifically in variants with a gain-of-function assay result. Variant class, residual protein function, developmental stage at treatment, predominant seizure type, background medication, and neurodevelopmental comorbidity all modify response. Syndrome-level enrolment can therefore obscure biologically distinct subgroups and dilute—or even completely hide—a real effect. Functional variant classification, performed prospectively and reported transparently, should increasingly be treated as an eligibility criterion rather than a post hoc covariate.

**Endpoints.** Countable motor seizure frequency is a necessary endpoint but an insufficient one. It does not capture non-motor and subtle seizures, which are systematically under-ascertained in exactly the populations where they matter most; it is insensitive to the developmental, behavioural, and communicative changes that families consistently rank above seizure count; and a median percentage reduction says nothing about durability or about the proportion achieving sustained freedom. Global impression scales and quality-of-life instruments partly address this but are vulnerable to unblinding and are rarely powered as primary outcomes. The most useful direction is hierarchical or co-primary endpoint structures that pair a seizure-count outcome with a prespecified, validated, caregiver-reported developmental or functional measure, together with time-to-event analyses of sustained seizure freedom and explicit reporting of response durability.

**Small samples, multiplicity, and safety.** Several of the most influential datasets in this review derive from cohorts of fewer than twenty patients. Small samples do not merely reduce power; they render effect estimates unstable, inflate the magnitude of any effect that does reach significance, and make subgroup differences essentially uninterpretable. They also cannot characterize uncommon adverse events, which is a material limitation for chronically administered drugs in children. Prespecified multiplicity control, pre-registered analysis plans, honest reporting of confidence intervals rather than point estimates, and pooled safety databases spanning related programmes would all improve inference without slowing development.

**Dose selection and target engagement.** Underpowered or abbreviated phase II dose-finding is, in the author’s view, the most under-recognized cause of late-stage failure in this field. Where no validated pharmacodynamic biomarker exists, exposure–response relationships are inferred from small studies and central nervous system target engagement is assumed rather than measured, so a negative phase III result cannot distinguish a wrong target from an inadequate dose. The vormatrigine POWER1 design illustrates the difficulty: a fixed escalation from 20 mg to 30 mg midway through a 12-week trial confounds dose with time, and a greater effect during the second half is compatible with a dose effect, a duration effect, or both. Transcranial magnetic stimulation measures of cortical excitability, quantitative EEG signatures, photosensitivity paradigms, and receptor-occupancy imaging are all available in principle and are used too rarely in practice, in my opinion.

**Translational models.** The acute electrical models that dominate screening—maximal electroshock and the 6 Hz assay—select efficiently for compounds that suppress ictogenesis in a normal brain, which is not the same as efficacy in a chronically epileptic, pharmacoresistant, developmentally abnormal one. Chronic and pharmacoresistant models improve prediction but are slower and less standardized. Genetic models are closer to the target biology yet are strain- and variant-specific, and behavioural or developmental outcomes in these models are far less often reported than seizure counts—a gap that matters precisely because developmental outcome is the endpoint that DEE families care most about.

**Reading a negative trial.** Programmes fail for at least six distinguishable reasons and misunderstanding them hurts the field. *Target failure* means the biology was wrong; *molecule failure* means the target was right but the compound lacked potency, selectivity, or brain penetration; *dose failure* means the compound never reached the necessary exposure; *trial-design failure* means the study could not have detected a true effect; *population-selection failure* means the effect exists in a subgroup that was diluted; and *endpoint failure* means the benefit was real but unmeasured. Bumetanide in neonatal seizures is a plausible instance of combined target and molecule failure, given both the questioned NKCC1 premise and poor brain penetration; ivermectin failed on molecule grounds; soticlestat’s narrowly missed phase III primaries with positive secondary measures are more consistent with endpoint or design limitations than with refutation of the cholesterol-24-hydroxylase hypothesis; and padsevonil remains genuinely ambiguous between dose and target failure. The vormatrigine POWER1 result should likewise not be read as evidence against functional-state sodium-channel modulation as a strategy or even vormatrigine as a potential new ASM—particularly given that the same strategy, in relutrigine, is supported by randomized data in a biomarker-defined population. In addition, dose–response analysis of POWER1 reported a better response in the 30 mg part of the trial, consistent with dose-effect sizes, perhaps even encouraging a redesign of the paused POWER2 trial to explore higher doses, rather than scraping out a promising drug entirely. Such lessons in clinical trials addressing epilepsy are still being learned the hard way and this review is intended exactly to provide a guide for a faster way forward in our field. 

**Priorities for future development.** Several changes would, in my view, make market entry for new antiseizure drugs both faster and more rational, and would also serve academic groups working with experimental compounds. Enrichment strategies should be biological rather than syndromic, using functional variant classification and, where available, electrophysiological or molecular biomarkers to define who is randomized. Adaptive and seamless phase II/III designs, with prespecified dose-selection rules, would reduce the number of programmes that enter confirmatory trials at the wrong dose. Rigorous natural-history cohorts—already being assembled in several DEEs—provide external comparators that make uncontrolled data interpretable and are indispensable for any disease-modification claim. Blinded follow-up should be extended beyond the twelve weeks conventional in adjunctive trials, since durability is a clinical question and not merely a regulatory one. Seizure ascertainment should be standardized and, increasingly, instrumented, using wearable and EEG-based detection to reduce diary noise. Endpoints should be co-developed with families to address the issues they care about the most. Multiplicity should be controlled in advance rather than explained afterwards. Negative and discontinued trials should be published in full, promptly and in the peer-reviewed literature, since the current asymmetry—in which positive interim results appear at congresses within months while negative studies appear years later or not at all—systematically biases the evidence base that this review and others must rely on, also hindering the lessons learned from failure trials. An amazing example of this is the rise in evidence-based thrombectomy for acute ischemic stroke, which after many years of failure trials, finally addressed the issue’s negative studies, and today is common practice. Finally, sponsors and congresses would serve the field well by reporting open-label data with full denominators and attrition, and by separating registrational from exploratory analyses explicitly in their abstracts.

**Clinical positioning.** None of this argues against the pipeline; it argues for reading it accurately. The realistic expectation is that emerging agents will complement, refine, and expand conventional antiseizure therapy rather than replace it. Their eventual position will be determined by comparative efficacy and tolerability, durability of response, accessibility and cost, monitoring burden, drug–drug interaction profile, and real-world effectiveness in unselected populations—none of which is established by the data currently available for most compounds discussed here.

## 14. Discussion and Future Directions

Several overarching themes emerge from the current development pipeline. First, precision and genotype-matched therapy has moved from aspiration to active clinical testing, most visibly in the DEEs, where agents are increasingly matched to specific channelopathies or gene defects—relutrigine and NBI-921352 for sodium-channel DEEs, radiprodil for *GRIN* variants, zorevunersen and ETX-101 for *SCN1A*-DS, and alogabat for Angelman syndrome [4,5]. Second, mechanistic novelty is genuine, even where clinical confirmation is not yet complete: K_V_7 opening, cholesterol-24-hydroxylase inhibition, selective 5-HT_2C_ agonism, persistent-current selectivity, and gene-directed modalities all represent departures from the conventional repertoire. Third, multimodal pharmacology—exemplified by cenobamate—offers a plausible route to superior efficacy, consistent with the view that epileptic networks are better addressed by agents acting on multiple nodes than by single-target drugs [30,49]. Fourth, the field is beginning to target epileptogenesis itself, with the first clinical proof of principle for prevention now in hand [46]. Although closely related, precision medicine, disease modification, and antiepileptogenesis represent distinct therapeutic objectives. Precision therapies are directed toward specific molecular causes of epilepsy, whereas disease-modifying interventions alter long-term disease trajectory, and antiepileptogenic therapies aim to prevent epilepsy before its clinical onset.

**Figure 6:**
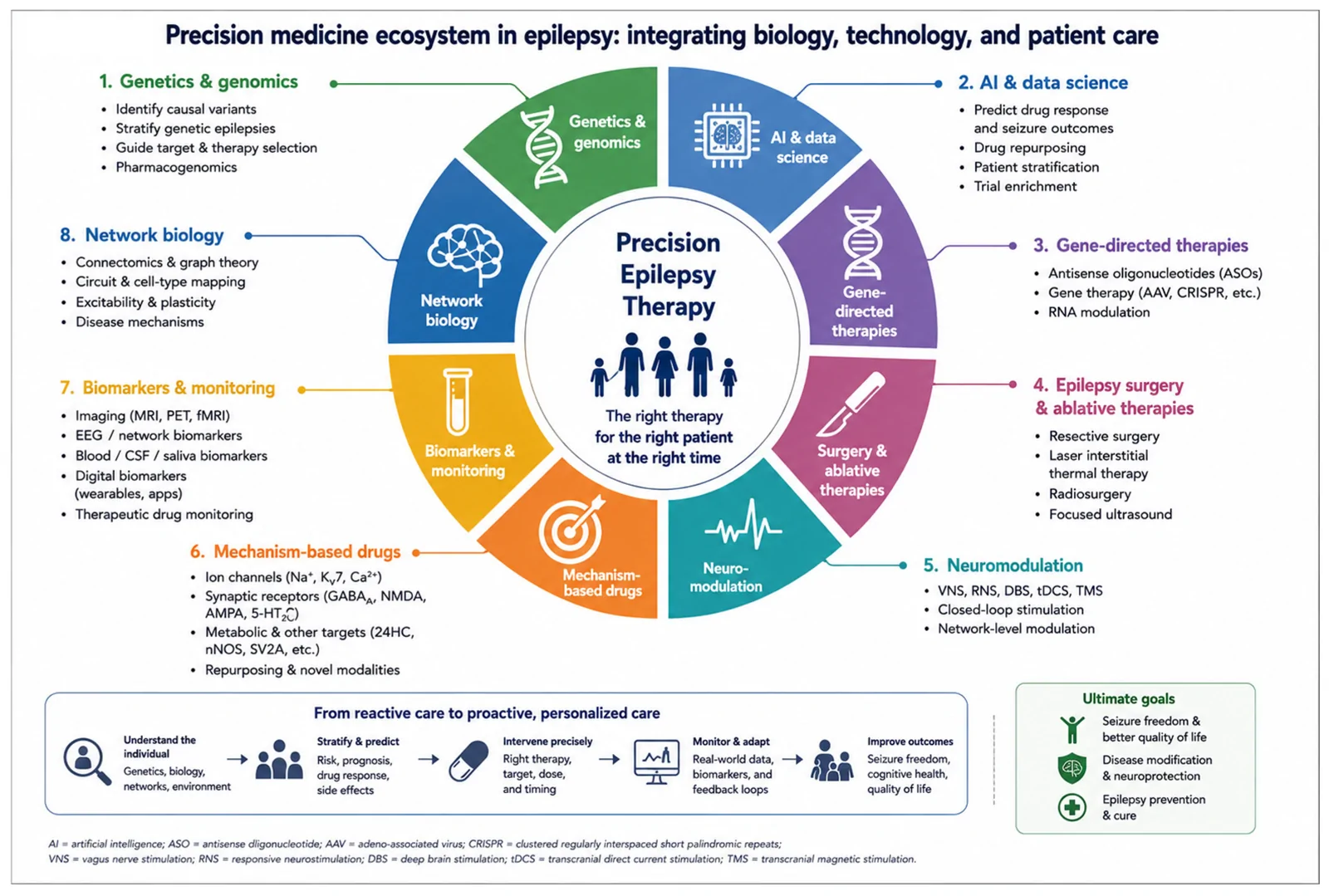
Precision medicine ecosystem in epilepsy: Integrating biology, technology, and patient-centred care. Integrated framework illustrating the multidisciplinary components required to implement precision medicine in epilepsy, including genetics and genomics, biomarkers, artificial intelligence, network biology, mechanism-based pharmacology, gene-directed therapies, neuromodulation, and epilepsy surgery. Together, these complementary domains are intended to support individualized treatment selection, prediction of therapeutic response, optimization of long-term outcomes, and the development of disease-modifying strategies. The figure depicts an aspirational framework; several of its components, notably validated predictive biomarkers and artificial intelligence-guided treatment selection, are not yet established in routine practice.

Important challenges remain. For many phase II agents, adequately powered placebo-controlled trials have not been completed; for several of the most prominent, the pivotal data exist only as congress presentations or interim analyses. The translational gap and the appraisal in the Section Critical Appraisal of Contemporary Antiseizure-Drug Development together indicate considerable caution in interpreting early signals [4]. Long-term efficacy and safety, particularly for gene and cell therapies, require extended follow-up; durability of effect, delivery, immunogenicity, and cost-effectiveness are unresolved. Pediatric and geriatric populations, in whom much of this pipeline will ultimately be used, are understudied. Comparative effectiveness data—direct head-to-head trials and effects on cognition, behaviour, comorbidities, and quality of life rather than seizure counts alone—are largely absent [31,53]. Predictive biomarkers of response would substantially improve trial efficiency and clinical targeting but remain underdeveloped. Future research should prioritize biomarker discovery, comparative-effectiveness studies, and outcome measures extending beyond seizure frequency to cognition, behaviour, quality of life, and neurodevelopment. Adaptive trial designs, digital biomarkers, and genotype-enriched enrollment strategies may improve the efficiency of clinical development, while long-term observational studies will be essential to determine whether emerging precision therapies truly modify disease trajectory rather than simply suppress seizures (Figure 6).

These emerging pharmacological strategies should be viewed as complementary rather than competitive with advanced epilepsy therapies. Precision pharmacology may substantially reduce seizure burden and delay referral for invasive treatment in selected genetic epilepsies, whereas epilepsy surgery and neuromodulation will likely remain essential for patients with focal structural epilepsies or persistent drug resistance. Future management will increasingly rely on integrating pharmacological, surgical, neuromodulatory, and genetic approaches within individualized treatment pathways rather than considering them mutually exclusive options.

For clinicians, these developments have immediate implications. Several agents with differentiated mechanisms—azetukalner for focal epilepsy, relutrigine for sodium-channel developmental and epileptic encephalopathies, bexicaserin for developmental and epileptic encephalopathies, and zorevunersen for Dravet syndrome—may enter clinical practice within the next few years, contingent on regulatory review and, in several cases, on confirmatory trials that have not been reported at the time of writing. None are currently approved for these indications, and none should be described to families as an established therapy. More broadly, the convergence of molecular diagnosis, biomarker-guided treatment selection, and disease-specific therapeutics is reshaping epilepsy management from empirical antiseizure therapy toward mechanism-based precision medicine.

## 15. Conclusions

The contemporary antiseizure medication pipeline represents one of the most significant transformations in epilepsy therapeutics since the introduction of second-generation antiseizure medications more than two decades ago. Whereas previous generations largely refined established pharmacological mechanisms, the current pipeline is characterized by genuine mechanistic innovation, precision targeting, and the first clinically testable strategies aimed at disease modification. Functional-state-selective sodium-channel modulation has moved from concept to late-stage clinical testing: relutrigine, on the strength of the EMBOLD randomized data and an accepted Priority-Review NDA, may become the first targeted therapy for *SCN2A*/*SCN8A*-DEEs, while vormatrigine’s controlled-trial setback tempers expectations for the same approach in common epilepsy [18,25]. Around them, K_V_7 openers (azetukalner), the dual-mechanism benchmark cenobamate, cholesterol-24-hydroxylase inhibition (soticlestat), serotonergic (bexicaserin) and glutamatergic (radiprodil) precision agents, subtype-selective GABA_A_ and sodium-channel modulators, and gene-directed therapies (zorevunersen, elsunersen) collectively signal a genuine diversification of epilepsy therapeutics. It bears repeating that much of the supporting evidence remains preliminary: several of the efficacy figures cited throughout derive from congress presentations, interim analyses, or open-label extensions and have not been published in full. What the field needs next is not more early signals but randomized confirmation, validated pharmacodynamic biomarkers, rigorous dose selection, endpoints that matter to patients and families, and the transparent publication of negative as well as positive results [4,7]. Although important translational challenges remain, the field is progressing, perhaps for the first time, from empiric seizure suppression toward mechanism-based, genotype-informed therapy. True disease modification remains an aspirational but—for the first time—an increasingly testable goal rather than an achieved one. This transition is redefining both the scientific framework of antiseizure drug development and the future clinical management of epilepsy.

## Figures and Tables

**Table 1:** Antiseizure medications with novel or differentiated mechanisms in advanced clinical development (phase II–III), grouped by molecular target.

Drug (Code)	Target/Mechanism of Action	Main Indication (s)	Phase	Status/Key Data	Evidence Source (s)
**Voltage-gated sodium channels**					
Relutrigine (PRAX-562)	Preferential persistent Na+ current inhibitor; functional-state-selective	*SCN2A*- and *SCN8A*-DEE	II (registr.)	EMBOLD positive (~46% and ~53% placebo-adjusted; AES 2024–2025); NDA under FDA Priority Review (PDUFA 27 September 2026); BTD/ODD/RPDD	Mechanism/preclinical (PR preclinical): [21]. Efficacy: [17,22,23] (congress presentations, incl. interim analysis stopped early; full publication pending). Trial status: [24] (registry, acc. 30 June 2026). Designations/PDUFA: [25] (regulatory record, acc. 30 June 2026).
Vormatrigine (PRAX-628)	Functionally selective Na+ channel modulator (hyperexcitable state); also Nav1.7/1.8	Adult focal onset and generalized	II/III	RADIANT 56.3% (open-label; AES 2025, IEC 2025); POWER1 missed primary (NCT06999902); POWER2 paused	Open-label efficacy: (congress; publication pending) [26]. Controlled trial and status: [18] (registry, acc. 30 June 2026). Mechanism: sponsor-reported; no PR pharmacology paper.
NBI-921352 (XEN901)	Selective Nav1.6 inhibitor	*SCN8A*-DEE; adult focal	II	First-in-class Nav1.6-selective	Mechanism/preclinical: (PR preclinical) [27]. Stage: [5] (PR review). No PR clinical efficacy data.
Carisbamate (YKP509)	Na^+^ channel block + T-type Ca^2+^ + AMPA/NMDA attenuation	Lennox–Gastaut syndrome	III	Phase 3 (drop seizures); FDA Orphan Drug	Mechanism and stage: (PR reviews) [5,15]. Phase III status and ODD: registry/regulatory records, acc. 30 June 2026. No PR phase III efficacy data.
Cenobamate (YKP3089) ^†^	Persistent Na+ inhibition + GABA-A PAM (non-BZD site)	Focal (approved)	Approved	Benchmark; highest seizure-freedom probability in 2026 network meta-analysis	RCTs: (PR randomized) [28,29]. OLE: [6] (PR open-label). Mechanism: [30] (PR review); secondary ion-channel actions [11] (PR pharmacology). Ranking: [19,31] (PR indirect comparison).
**Potassium channels**					
Azetukalner (XEN1101)	Selective Kv7.2/7.3 (KCNQ) channel opener	Adult focal; primary generalized; MDD	III	X-TOLE2 positive (AAN 2026); OLE ~90% at 48 mo (AAN 2026); FDA filing planned	Phase IIb: (PR randomized) [32]. Phase III X-TOLE2 and 48-month OLE: [7,33] (congress; interim; publication pending). Selectivity: sponsor in vitro data; class pharmacology [14] (PR review).
BHV-7000	Kv7.2/7.3 modulator	*KCNQ2*-DEE; focal	II/III	Restores KCNQ2-variant current in vitro	Mechanism and stage: (PR review); in vitro variant-rescue data sponsor-reported. No PR clinical efficacy data [5].
XEN496 (retigabine)	Kv7 opener (pediatric formulation)	*KCNQ2*-DEE	III	Precision repurposing of retigabine	Rationale and stage: (PR review); class pharmacology [12,13] (PR). Registry status acc. 30 June 2026 [5].
AUT-00206	Kv3.1/3.2 modulator	Fragile X syndrome	II	Enhances interneuron function	Mechanism and stage: (PR review). Preclinical/pharmacodynamic evidence only [5].
**GABA-A/GABA-B receptors**					
Darigabat (CVL-865)	α2/3/5-selective GABA-A PAM (partial agonist)	Adult focal	II	AbbVie; proof-of-concept in photosensitivity	Preclinical: (PR preclinical) [34]. Photosensitivity proof-of-concept and stage: [5,8] (PR reviews). No PR phase II efficacy data.
Alogabat (RG-7816)	α5-selective GABA-A PAM	Angelman syndrome	II	Precision rationale (α5 subunit loss)	Rationale and stage: (PR reviews) [5,8]. No PR efficacy data.
AZD7325 (BAER-101)	α2/α3-selective GABA-A PAM	Fragile X syndrome	II	—	Stage and mechanism: (PR reviews) [5,8]. No PR efficacy data.
Gaboxadol (OV101)	Extrasynaptic δ-GABA-A agonist (tonic inhibition)	Angelman; fragile X	I/II	—	Mechanism and stage: (PR reviews). No PR seizure-efficacy data [5,8].
Ganaxolone ^†^	Neurosteroid GABA-A PAM (synaptic + extrasynaptic)	Refractory SE (IV); approved CDKL5	II/III	Useful where synaptic receptors internalize	Mechanism and rationale: (PR review/preclinical) [35]. Stage: [5] (PR review). Approved in CDKL5 deficiency disorder; status-epilepticus programme investigational.
Alprazolam inhaled (AZ-002)	GABA-A PAM (inhaled, Staccato)	Cluster-seizure rescue	III	Rapid seizure termination	Rationale and stage: (PR review); registry status acc. 30 June 2026 [5].
Basmisanil (RG-1662)	α5-selective GABA-A NAM (inverse agonist)	Angelman; Dup15q	II	Procognitive	Mechanism and stage: (PR reviews). Procognitive rationale; no PR seizure-efficacy data [5,8].
Arbaclofen	GABA-B receptor agonist	Fragile X syndrome	III	Normalizes E:I balance in models	Mechanism and preclinical rationale: (PR review). No PR seizure-efficacy data [5].
**Glutamate receptors**					
Radiprodil	GluN2B-selective NMDA NAM	*GRIN* gain-of-function variants	II	Precision for *GRIN*-DEE	Rationale and stage: (PR review) [5]. Open-label phase Ib/2a and pivotal programme: [36] (registry, acc. 30 June 2026). Efficacy data not yet peer-reviewed.
Basimglurant	mGlu5 antagonist	TSC; fragile X	II	Also tested in depression	Mechanism and stage: (PR review) [5].
Acamprosate ^†^	mGlu5/NMDA modulator	Fragile X syndrome	III	Approved for alcohol dependence	Stage: (PR review). Approved in another indication; no PR epilepsy-efficacy data [5].
JNJ-40411813 (ADX71149)	mGlu2 PAM	Focal (LEV/BRV suboptimal)	II	Potentiates levetiracetam (6 Hz model)	Preclinical synergy and stage: (PR review) [5].
JBPOS-0101	mGlu1/4/7 antagonist	DEEs; refractory SE	II	Active in BZD-resistant SE models	Preclinical activity and stage: (PR review) [5].
Selurampanel	AMPA receptor antagonist	Focal	II	Also studied in migraine/tinnitus	Stage: (PR review). Class precedent (perampanel): [4,5] (PR review).
**Serotonin (5-HT) receptors**					
Bexicaserin (LP352)	Selective 5-HT2C superagonist	DEEs (Dravet, LGS, others)	III (DEEp)	PACIFIC ~60% vs. 17%); OLE ~59% at 52 wk; BTD; Lundbeck	Phase Ib/2a: (PR randomized) [37]. OLE: [38] (congress; publication pending). Phase III and designations: [37,38]; registry/regulatory records, acc. 30 June 2026.
Lorcaserin (EPX-200) ^†^	5-HT2C agonist	Dravet syndrome	II/III	Repurposed (former anti-obesity)	Stage: (PR review). Repurposed agent; no PR randomized epilepsy data [5].
Clemizole (EPX-100)	5-HT receptor modulation	Dravet syndrome	II	Identified in zebrafish model	Discovery and stage: (PR review) [5].
Psilocybin (NM-1001)	5-HT2A agonist	Fragile X syndrome	II	Oral microdose	Stage: (PR review). No PR seizure-efficacy data [5].
**Other/novel small-molecule mechanisms**					
Soticlestat (TAK-935)	Cholesterol-24-hydroxylase (CYP46A1) inhibitor	Dravet; Lennox–Gastaut	III	SKYLINE/SKYWAY narrowly missed primary; positive secondaries	Mechanism: (PR review) [39]. Phase II ELEKTRA: [40] (PR randomized). Phase III SKYLINE/SKYWAY: [39,40] and registry records, acc. 30 June 2026; full phase III publication pending.
Vatiquinone (PTC-743)	15-lipoxygenase inhibitor (oxidative stress)	Mitochondrial epilepsy	II/III	Also in Friedreich ataxia	Mechanism and stage: (PR review) [5].
Sodium selenate	Protein-phosphatase-2A activator (disease-modifying)	Temporal lobe epilepsy	II	Disease-modification hypothesis	Hypothesis and stage: [5] (PR review). Preclinical rationale only; clinical disease modification not demonstrated.
Zatolmilast (BPN14770)	PDE4D allosteric inhibitor	Fragile X syndrome	III	Cognitive/language signal	Mechanism and stage: [5] (PR review). Cognitive/language signal not a seizure endpoint.
SPN-817	Acetylcholinesterase inhibitor (huperzine A)	Focal impaired-awareness	II	Extended-release	Mechanism and stage: [5] (PR review).
NRP2945	Neural-regeneration peptide; increases GABA-A subunit expression	LGS; absence epilepsy	II	Antiseizure + antiepileptogenic (preclinical)	Preclinical only: [5] (PR review). Antiepileptogenic claim is preclinical.
Blarcamesine (ANAVEX2-73)	Sigma-1 receptor agonist	Rett; infantile spasms; fragile X	I–III	Also in Alzheimer/Parkinson dementia	Mechanism and stage: [5] (PR review).
Ataluren ^†^	Premature stop-codon read-through	Nonsense Dravet; CDKL5	II	Negative small phase 2	Negative small phase II: [5] (PR review).
2-Deoxy-glucose	Glycolytic inhibitor	Epilepsy (unspecified)	II	—	Stage: [5] (PR review).
**Anti-inflammatory/metabolic/microbiome**					
Anakinra ^†^	IL-1 receptor antagonist	FIRES	Case series	Mixed results; tocilizumab alternative	Case-series evidence only: [5] (PR review).
Belnacasan (VX-765)	Caspase-1/IL-1β inhibitor	Focal	II	Modest efficacy	Stage and modest efficacy: [5] (PR review).
Tricaprilin	C8 medium-chain triglyceride (ketogenic)	Infantile spasms	I	—	Stage: [5] (PR review).
*Lactobacillus* probiotic	Gut-microbiota modulation	Refractory childhood epilepsy	II	Gut–brain axis	Stage: [5] (PR review). Gut–brain rationale; no PR randomized data.
Fecal microbiota suspension	Gut-microbiota modulation	Epilepsy (unspecified)	II/III	—	Stage: [5] (PR review). No PR randomized data.

ASO, antisense oligonucleotide; BTD, breakthrough therapy designation; BZD, benzodiazepine; CDKL5, cyclin-dependent kinase-like 5; DEE, developmental and epileptic encephalopathy; E:I, excitatory:inhibitory; FIRES, febrile infection-related epilepsy syndrome; LGS, Lennox–Gastaut syndrome; MDD, major depressive disorder; NAM, negative allosteric modulator; ODD, orphan drug designation; PAM, positive allosteric modulator; RPDD, rare pediatric disease designation; SE, status epilepticus; TSC, tuberous sclerosis complex. ^†^ Agent already approved in this or another indication, included for mechanistic context. Trial figures are sourced to peer-reviewed publications where available and otherwise to congress presentations and to regulatory agencies; they are not based on company press releases. **Evidence-source column:** Each row identifies the source supporting the mechanistic statement, the development phase, the main efficacy or safety result, and the trial or regulatory status, together with the provenance class of that source. PR, peer-reviewed; OLE, open-label extension; acc., accessed. Provenance classes are defined in Section 2. Congress-reported and interim findings are preliminary and may change on full publication; open-label extension results are subject to enrichment, attrition, and ascertainment bias and are not comparable with placebo-adjusted estimates. Registry and regulatory statuses are current only to the access date shown. Full registry records, with trial titles, registration numbers, and direct URLs, are given in the reference list.

**Table 2:** Gene-directed and cell therapies in clinical development for epilepsy.

Therapy (Code)	Modality	Target/Mechanism	Indication	Phase	Status	Evidence Source (s)
Zorevunersen (STK-001)	ASO (TANGO)	↑ *SCN1A*	Dravet syndrome	III	Biogen-partnered; BTD; phase 1/2a in NEJM 2026; EMPEROR readout ~2027	Preclinical (TANGO): [42] (PR preclinical). Phase I/IIa and extensions: [20] (PR open-label, uncontrolled). Phase III EMPEROR status: [43] (registry, acc. 30 June 2026). Disease modification not established.
Elsunersen (PRAX-222)	ASO	↓ *SCN2A* (gain-of-function)	*SCN2A*-DEE	I/II	ODD/RPD (FDA); ODD/PRIME (EMA)	Rationale and stage: [24] (registry, acc. 30 June 2026); designations per FDA/EMA records, acc. 30 June 2026. No clinical efficacy data published.
ETX-101	AAV gene therapy	↑ *SCN1A* in GABAergic interneurons	*SCN1A*-Dravet	I/II	Regulatory element + transcription factor	Mechanism and stage: [5] (PR review); registry status acc. 30 June 2026. First-in-human; no efficacy data published.
NRTX-1001	Regenerative cell therapy	GABA-releasing interneuron transplant	Unilateral mesial TLE + HS	I/II	—	Mechanism and stage: [5] (PR review); registry status acc. 30 June 2026. First-in-human.
Lentiviral Kv-channel therapy	Gene therapy	Engineered K+ channel delivery	Refractory focal epilepsy	I	—	Mechanism and stage: [5] (PR review). Preclinical/first-in-human.
AMT-260	AAV gene therapy	↓ GluK2 kainate-receptor subunit	Mesial temporal lobe epilepsy	Precl./I	—	Mechanism and stage: [5] (PR review). Preclinical/first-in-human.
ION-582	ASO	*UBE3A* reactivation (↓ UBE3A-ATS)	Angelman syndrome	II	—	Mechanism and stage: [5] (PR review). Seizure outcomes not primary.
GTX-102	ASO	*UBE3A* reactivation (paternal allele)	Angelman syndrome	II	—	Mechanism and stage: [5] (PR review). Seizure outcomes not primary.

↑, increase; ↓, decrease; AAV, adeno-associated virus; ASO, antisense oligonucleotide; HS, hippocampal sclerosis; TANGO, targeted augmentation of nuclear gene output; TLE, temporal lobe epilepsy; PR, peer-reviewed; acc., accessed. All programmes in this table are at first-in-human or early clinical stage except zorevunersen; none have demonstrated clinical disease modification. Evidence-source entries identify the source supporting the mechanism, phase, and status of each row and the provenance class of that source (Section 2); registry and regulatory statuses are current only to the access date shown.

**Table 3:** Mechanistically promising candidates that recently failed or were discontinued in advanced development.

Drug (Code)	Mechanism of Action	Indication	Phase Reached	Outcome	Evidence Source (s)
Padsevonil	SV2A/B/C modulation + GABA-A BZD-site partial agonism	Drug-resistant focal	III	Failed primary endpoints; discontinued	Phase IIb/III: [48] (PR randomized). Interpretation: dose versus target failure unresolved (Section Critical Appraisal of Contemporary Antiseizure-Drug Development).
Brexanolone (allopregnanolone)	Neurosteroid GABA-A PAM (synaptic + extrasynaptic)	Super-refractory SE	III	Failed phase 3; discontinued	Outcome: [5] (PR review). Open-label success not reproduced under blinding; molecule versus design failure not distinguished.
Bumetanide	NKCC1 cotransporter inhibitor	Neonatal seizures; autism	II/III	Inconsistent efficacy; ototoxicity risk	Outcome and mechanistic critique: [5] (PR review). Consistent with combined target and molecule (brain-penetration) failure.
Ivermectin (EQU-001)	Non-selective GABA-A PAM	Focal onset	II	Modest effect; development halted	Outcome: [5] (PR review). Consistent with molecule failure (poor blood–brain-barrier penetration).

BZD, benzodiazepine; NKCC1, Na-K-2Cl cotransporter 1; PAM, positive allosteric modulator; SE, status epilepticus; SV2, synaptic vesicle glycoprotein 2; PR, peer-reviewed. Inclusion in this table indicates that a programme did not meet its primary endpoint or was discontinued; it does not establish that the underlying molecular target is invalid. Target, molecule, dose, design, population, and endpoint failures are distinguished in the Section Critical Appraisal of Contemporary Antiseizure-Drug Development.

## Data Availability

No new data were created. All sources analyzed are publicly available and are cited in the reference list, including direct URLs and access dates for clinical-trial registry and regulatory records.
